# Exploring the Role of Extracellular Vesicles in Pancreatic and Hepatobiliary Cancers: Advances Through Artificial Intelligence

**DOI:** 10.3390/ijms27031524

**Published:** 2026-02-04

**Authors:** Eleni Myrto Trifylli, Athanasios Angelakis, Sotirios P. Fortis, Anastasios G. Kriebardis, Nikolaos Papadopoulos, Evangelos Koustas, Panagiotis Sarantis, Michalis V. Karamouzis, Spilios Manolakopoulos, Melanie Deutsch

**Affiliations:** 1 Gastrointestinal-Liver Unit, 2nd Department of Internal Medicine, General Hospital of Athens “Hippocratio”, National and Kapodistrian University of Athens, 114 Vas Sofias, 11527 Athens, Greece; nipapmed@gmail.com (N.P.); smanolak@med.uoa.gr (S.M.); meladeut@gmail.com (M.D.); 2 Institute of Molecular Medicine and Biomedical Research (IMBE), 11527 Athens, Greece; panayotissarantis@gmail.com (P.S.); mkaramouz@med.uoa.gr (M.V.K.); 3 Laboratory of Reliability and Quality Control in Laboratory Hematology (HemQcR), Department of Biomedical Sciences, Section of Medical Laboratories, School of Health & Caring Sciences, University of West Attica (UniWA), Ag. Spyridonos Str., 12243 Egaleo, Greece; sfortis@uniwa.gr (S.P.F.); akrieb@uniwa.gr (A.G.K.); 4 Department of Epidemiology and Data Science, Amsterdam University Medical Center, 1105 Amsterdam, AZ, The Netherlands; a.angelakis@amsterdamumc.nl; 5 Oncology Department, General Hospital Evangelismos, 10676 Athens, Greece; vangkoustas@gmail.com; 6 Academic Department of Internal Medicine, General and Oncology Hospital “Agioi Anargyroi”, National and Kapodistrian University of Athens, Timiou Stavrou 14, 14564 Kifisia, Greece

**Keywords:** extracellular vesicles, liquid-biopsy, nanotechnology, gastrointestinal cancer, non-invasive tests, biomarkers, EV-engineering, artificial intelligence, data science, machine-learning, deep learning, drug-delivery, exosomes, oncosomes

## Abstract

Gastrointestinal (GI) cancers constitute an umbrella term for a wide variety of malignancies that are located in the digestive tract (esophageal, gastric, small and large intestine, anus, liver, gallbladder, and pancreas), with 25% of total cancers and 35% of cancer-related deaths being attributed to them. An alarming trend of rising GI malignancy diagnoses, especially in younger age groups, underscores the need for discoveries in liquid-based biomarkers that facilitate both early detection and optimal disease management. Extracellular vesicles (EVs) not only constitute promising nano-sized biomarkers, but also, via bioengineering, have shown a great therapeutic potential, with artificial intelligence (AI) revolutionizing their research via the selection of the best biomarkers from omics, the recognition of pathophysiological patterns, and facilitating a faster drug-development via AI-driven EV engineering, drug delivery modeling, and target identification. In this review, we will provide a clear insight into the implementation of AI methodologies in EV-based biomarker discovery and therapeutics for pancreatic and hepatobiliary cancer.

## 1. Introduction

Gastrointestinal (GI) cancers are an umbrella term that includes all the types of malignancies affecting the digestive tract and the accessory digestive organs, with the major GI cancers including: hepatocellular carcinoma (HCC), cholangiocarcinoma (CCA), gallbladder cancer (GC), and pancreatic cancer (PC). GI malignancies account for 23.9% of all cancers and 33.2% of all deaths globally, based on the Current Global Burden 2022, whereas the global incidence is estimated to be increasing by approximately 12.8% until 2025 [1]. There are some geographical trends of GI cancer incidence, with upper GI malignancies being highly diagnosed in Asian (70% of the cases) countries, as well as in those with a higher human development index (HDI), starting with Europe. Likewise, high-HDI and Western countries present a higher incidence of CRC and PC diagnosis [1,2]. Despite all the efforts for national and international screening efforts, diagnostic tools, and new therapeutic modalities, GI cancer remains a major threat [1]. Development of novel liquid-biopsy tools for GI cancer early diagnosis, prognosis, prediction, and monitoring is in the spotlight of current studies. Among several emerging molecules that are studied, extracellular vesicles (EVs) are gaining widespread recognition for their potential use as biomarkers in oncology [3,4].

EVs are double-membraned nanoparticles between 50 and 1000 nm, subclassified into exosomes (50–150 nm), microvesicles (150–1000 nm), and apoptotic bodies (above 1000 nm), resulting from inward and outward membrane budding and cell apoptosis, respectively. However, their heterogeneity is not only in their size and biogenesis, but also in their embedded cargoes, which have a crucial role in intercellular communication between the parental and recipient cells. Their abundance in several body fluids, as well as their dynamic character as biomarkers that reflect the real-time modifications at a cellular level, make them promising tools for diagnosis, disease prognosis, and patient stratification [5]. Profiling of EVs’ origin and cargoes can indicate the origin of the parental cell, but also facilitate the identification of several GI cancer signatures that can help in early diagnosis, prognosis, and treatment monitoring [6].

The integration of AI in GI cancer research is considered crucial, as it can significantly accelerate not only the identification of novel biomarkers but also the discovery of new druggable targets, assisting in drug design and delivery, as well as toxicity prediction, which will potentially lead to the substantial shortening of the timeline between pre-clinical to clinical phases [7]. In this review, we explore the emerging applications of AI in facilitating the use of EVs for biomarker discovery and therapeutic innovations in pancreatic and hepatobiliary cancer.

## 2. Biology and Oncological Role of Extracellular Vesicles

### 2.1. EV Biogenesis and Intercellular Communication

These nano-sized vesicles are characterized by a high degree of heterogeneity, resulting from their various cell/tissue origin, the mechanism of their biogenesis (size variation), and the type of embedded cargoes inside their double phospholipid membrane. The variety of cargoes is wide, including non-coding/coding RNA molecules, DNA molecules, lipids, proteins, receptors, autophagosomes, mitochondrial DNA, and several metabolites that have key roles in physiological and pathophysiological procedures, as their uptake by recipient cells can significantly alter their functionality via inducing several signaling pathways [8]. The distance between the parental and the recipient cells is crucial for the mechanism of intercellular communication, including (i) paracrine, when the recipient cell is in the vicinity of the parental, (ii) autocrine, when the parental and the recipient cells are identical, and (iii) endocrine, when the distance between parental and recipient cell is long, and EVs reach the latter through blood circulation. Moreover, cross-talk between parental and recipient cells is mediated via several mechanisms, such as micropinocytosis, clathrin-mediated, caveolin-mediated, and lipid-raft endocytosis, as well as ligand–receptor interaction and direct fusion with the cell membrane. However, the mechanisms of delivery and the uptake of EVs by recipient cells need to be further studied [8,9,10].

### 2.2. EV Formation Routes: Exosomes, Microvesicles, Apoptotic Bodies

Exosome (50–150 nm) biogenesis and the cargo sorting mechanism start with the inward budding of the cell membrane, in which several transmembrane proteins are internalized under the action of Endosomal Sorting Complex Required for Transport (ESCRT) 0-III complex, which regulates these endosomal pathways. ESCRT-0 (Vps27, Hse1) initially identifies these transmembrane proteins, based on the presence of one or more ubiquitin molecules that are covalently attached to their surface. Vps27 is bound to the endosomal membrane for the subsequent binding of ESCRT I-III. More particularly, ESCRT-I (Vps23, Vps28) is bound to ESCRT-0, and ESCRT-II (Vps25, Vps36) eventually to ESCRT-I, with ESCRT-II facilitating the recruitment of ESCRT-III (Vps20) similarly. Vesicle formation requires the contribution of ESCRT 0-I, with early endosomes being matured (late endosomes) with the inward invagination of late endosomal membrane leading to intraluminal vesicle (ILV) formation, which are further enclosed within the lumen of multivesicular bodies (MVBs) under the action of ESCRT-II and -III. At this point, maturation of MVBs requires deubiquitination under the action of Bro1/ALIX proteins of the ESCRT complex. MVBs may follow different “pathways” such as (i) fusion with the cell membrane for exosome release in the extracellular space, (ii) lysosomal degradation, as well as (iii) fusion with autophagosomes for degradation or fusion with the membrane for exosome exocytosis under the action of soluble NSF-attachment protein receptor (SNARE) complex proteins, with the Ca^2+^-regulated vesicle-associated membrane protein 7 (VAMP7), which is part of it, being required for the MVB fusion with cell-membrane. Once formed, MVBs follow one of three potential pathways: (i) fusion with lysosomes, leading to degradation of their contents; (ii) fusion with the plasma membrane, resulting in the release of exosomes into the extracellular space, a process facilitated by SNARE proteins; or (iii) fusion with autophagosomes to form amphisomes, which may subsequently undergo degradation or merge with the plasma membrane to release exosomes to extracellular space. The Rab family (small GTPases such as Rab 7,11,27a/b,35) is involved in vesicle trafficking for their fusion. Nevertheless, ILV cargo sorting is not only mediated via ESCRT machinery, as an ESCRT-independent mechanism of exosome biogenesis is also identified. Cooperation of ADP-ribosylation factor 6 (ARF6), which is a GTP-binding protein and syntenin, induces exosome biogenesis, as well as with ARF6 effector and the phospholipase D2, while after MBVs are formed, they are similarly either degraded in lysosomes or fused with the cell membrane for exosome release. Additionally, several markers that are identified in exosomes are Rab family, CD9, Alix, CD63, CD81, Tgs101, tetraspanin, and ceramide, which are molecules that are taking part in the biogenesis and endosomal vesicle trafficking [4,8,9,10,11,12,13,14].

Microvesicles (MVs) (150–1000 nm) or ectosomes constitute the medium-sized subcategory of EVs, with their biogenesis starting with the outward blebbing of the cell membrane, with the regulation being mediated by several proteins such as TSG101 (ESCRT-I complex component) and ARRDC1 under physiological oxygen conditions, whereas SNAREs and Rab-GTPases mediate the cargo recruitment under hypoxia. Cell membrane requires several rearrangements for its blebbing, which is a result of modifications in Ca +2 levels, as well as in protein and lipid molecules at the site of the buds, with the externalization of phosphatidylserine also being a key characteristic of this biogenetic pathway. Moreover, the selective incorporation of cargoes into MVs, such as vesicle-associated protein 3, RNA and DNA molecules, β1-integrin, as well as membrane type 1 matrix metalloproteinase and Major Histocompatibility Complex class I (MHC-I), is facilitated by ARF6, as the ARF6-regulated endosomal complex has a crucial role in selective “packaging” of specific cargoes inside MVs. However, it is also implicated in MV shedding from the cell membrane, with its inhibition resulting in the reduction of MV release [4,15,16,17].

Apoptotic vesicles—apoptotic bodies (above 1000 nm) constitute the largest EVs, resulting from the cell-apoptotic mechanism, a multi-step procedure that initially includes nuclear fragmentation, the condensation of chromatin, and the breakdown of cellular organelles that leads to the formation of membrane protrusions. These protrusions eventually form the apoptotic vesicles and their segments, the so-called apoptotic bodies [13].

### 2.3. Tumor-Derived EVs (tEVs) and Oncosomes in Cancer Biology

Tumor-derived extracellular vesicles, including oncosomes (100–400 nm) and large oncosomes (1000–10,000 nm), carry oncogenic proteins, RNAs, and signaling molecules that drive tumor growth, metastasis, immune modulation, and microenvironment remodeling. Large oncosomes, shed from migratory tumor and stromal cells, contain more abundant cargo and are detectable in tissues and circulation, making them key biomarkers for cancer progression [18,19,20,21,22,23,24,25,26,27,28,29,30,31,32,33].

Oncosomes (100–400 nm) are a specialized subtype of tEVs that are derived from malignant cells with similar biogenesis pathways to exosomes and MVs, depending on their size. Like with exosome biogenesis, the members of the SNARE protein complex, such as VAMP 1–3, have been identified in the biogenesis of tumor-derived exosomes. Meanwhile, Rab 27a (Rab family of small GTPases), which is implicated in vesicle trafficking and fusion with the cell membrane, has a key role in exosome release by metastatic tumor cells [18,19,20,21,22]. Moreover, there are tumor-derived MVs that originate from the outward blebbing of tumor cells in a similar way to non-tumor-derived ones [18]. At this point, it is to be noted that the condition of parental cells (malignant or physiological) is crucial for the type and quantity of the molecular cargoes inside the EVs. Interestingly, they can carry several tumor-related biomolecules with oncogenic behavior, such as mutant proteins (e.g., c-Met), oncogenes, growth factors (e.g., epidermal growth factor receptor vIII), coding or non-coding RNA molecules, etc. These EVs contain tumor-specific cargoes that can be utilized not only for cancer diagnosis but also for monitoring, which can be isolated from a wide variety of biological fluids. Similar to non-tumor-derived EVs, they can alter the functional state of the recipient cells, promoting tumorigenesis, proliferation, tumor growth and survival, drug resistance, as well as neoangiogenesis and metastatic dissemination (implicated in metastatic niche), tumor microenvironment (TME) modifications, and extracellular matrix remodeling (ECM). ECM and tumor migration are attributed to the EV-contained molecules such as metalloproteinases MMPs, while TME modification results from the release of tumor-derived EVs that contain tumor-growth factor β1 (TGF-β1), which directly interacts with fibroblasts, while in the case of neoangiogenesis, EV-contained VEGF interacts with endothelial cells. Additionally, a tumor-derived EV modulation of immune cells (e.g., T regulatory/cytotoxic cells) has also been reported, such as in the case of EV-TGF-β1 that interacts with several immune cells of TME, while drug resistance has been correlated with mutant DNA molecules embedded in tumor-derived EVs, implying an EV-mediated immunotolerance [23,24,25,26,27].

**Figure 1:**
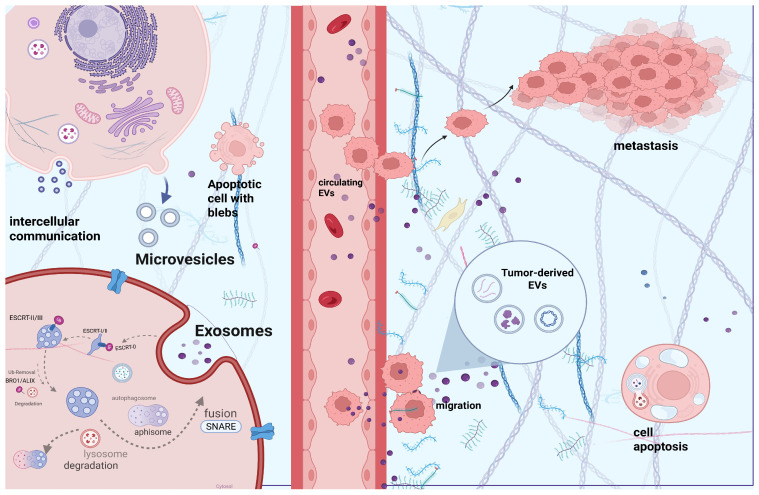
EV biogenesis and its role in intercellular communication within the TME. Microvesicles (150–1000 nm) or ectosomes constitute the medium-sized subcategory of EVs, with their biogenesis starting with the outward blebbing of the cell membrane, while apoptotic vesicles—apoptotic bodies (above 1000 nm)—constitute the largest EVs, resulting from the cell-apoptotic mechanism. Oncosomes (100–400 nm) are a specialized subtype of tumor-derived EVs that are derived from malignant cells, whereas large oncosomes (1000–10,000 nm) are distinct EVs with more potent oncogenic molecules as cargoes; their biogenesis resembles that of microvesicles, as both result from the direct shedding of the cell membrane, with ARF6 playing a key role. Exosomes’ (50–150 nm) biogenesis and cargo sorting mechanism start with the inward budding of the cell membrane under the action of Endosomal Sorting Complex Required for Transport (ESCRT) 0-III complex, leading to intraluminal vesicle formation within multivesicular bodies (MVBs), which fuse with the plasma membrane, resulting in the release of exosomes into the extracellular space. Likewise, amphisomes, resulting from autophagosomes and MVB fusion, may either fuse with the membrane or be degraded by lysosomes. Created in BioRender. Trifylli, E. (2026) https://BioRender.com/e99v625 (accessed on 28 January 2026). Agreement license UJ29AJYFRM.

Large oncosomes (LOs) are a distinct and non-synonymous term with oncosomes, with various disparities such as the diameter (1000–10,000 nm), with more potent oncogenic molecules as cargoes that are implicated in immune modulation, tumor progression, invasion, and TME modifications. Tumor cells secrete several sizes of EVs, with the control of shedding relatively unknown. Interestingly, miR-1227 overexpression has been shown to suppress SEC23A, leading to LO shedding. A higher amount of LOs-miR-1227 has been demonstrated in cancer (e.g., prostate cancer) compared to smaller EVs. These larger-diameter EVs have a greater capacity for accommodation of tumor-derived molecules [28], but also for highly metastasis-related cargoes, such as osteopontin, brain-derived neurotrophic factor (BDNF) [29], as well as C-X-C motif chemokine 12 (CXCL12) [28,29,30,31,32]. Meanwhile, it has been demonstrated that exosomes carry fewer miRNAs compared to LOs, which implies the utilization of the latter as a larger pool for biomarker discovery and cancer profiling. It has to be underlined that they are derived from highly migratory tumor cells (e.g., breast and prostate cancer, etc.) [28,33], while it has been primarily demonstrated that amoeboid cancer cells, as well as in cancer-associated fibroblasts (CAFs), present non-apoptotic blebs, which can eventually be shed from these cells, forming the so-called LOs [28]. LO biogenesis resembles that of MV’s, as both of them result from the direct shedding of the cell membrane, with ARF6 playing a key role in both cases. Interestingly, a correlation between the aggressiveness, the amount of the released LOs, and the rate of this particular blebbing has been demonstrated. The large size of LOs permits their observation by light microscopy, and they can be easily detected in formalin-fixed, paraffin-embedded sections of the malignant tumors or even in circulation after their purification from metastatic animal (mice) or human models. The origin of LOs can be various, as they are not only shed by cancer cells, but also from other cells that are located in TME (e.g., CAFs, endothelial cells, immune cells, etc.), leading to several modifications that facilitate migration and tumor progression [28,29,30,31]. Figure 1 presents a schematic overview of EV biogenesis and its role in intercellular communication within the TME, created in BioRender. Trifylli, E. (2026) https://BioRender.com/e99v625 (accessed on 28 January 2026). 

### 2.4. A Brief Overview of the Latest Guidelines Regarding the Nomenclature

According to the latest Minimal Information for Studies of Extracellular Vesicles (MISEV2023) guidelines published by the International Society for Extracellular Vesicles (ISEV) in 2024, the unsupported use of the term “exosomes” is discouraged and the umbrella term “extracellular particles (EPs)” is recommended, with non-vesicular components of EV isolates referred to as non-vesicular extracellular particles (NVEPs) [32].

In this review, however, we retain the term “exosomes” when it is used in the cited studies, for consistency with the original literature.

## 3. The Implication of EVs in Pancreatic and Hepatobiliary Cancer

### 3.1. Hepatocellular Carcinoma (HCC)

HCC constitutes the most commonly diagnosed primary liver cancer, accounting for 75–85% of cases. It is considered a major global health burden, as it is the third leading cause of malignancy-related mortality globally, based on GLOBOCAN 2020 statistics, while its incidence is expected to be over 55% higher between 2020 and 2040 due to population aging. There is a rising trend of HCC incidence in Western countries, which is mostly related to metabolic syndrome, with the leading cause being Metabolic Dysfunction-Associated Steatotic Liver Disease (MASLD). However, its incidence remains higher in Asia (East and Southeast) and Africa (Sub-Saharan), mainly due to chronic viral hepatitis B and C, despite antiviral therapy and HBV vaccination [34,35,36].

EVs have an emerging role in hepatocarcinogenesis, HCC development, and progression via their implication in intercellular communication and tumor immunosurveillance and cell-survival. HCC-derived EVs interact with several other types of recipient cells, including tumor microenvironment (TME) cellular components, altering the functional state of the latter and promoting HCC progression. Among the several types of EV-cargoes, tumor-promoting long, short, and circular non-coding RNA molecules or oncogenic proteins have a significant impact on HCC progression, promoting neovascularization, epithelial–mesenchymal transition (EMT), tumor invasion and migration, as well as metastatic dissemination [37].

- **EMT, pre-metastatic niche formation and metastatic dissemination**

Hepatocyte-derived EVs carry a diverse array of bioactive cargos that contribute to HCC progression. Notably, several EV subpopulations derived from HCC cells promote epithelial–mesenchymal transition (EMT) and metastatic dissemination, including those carrying CD147 [38], miR-92a-3p [39], miR-21 [40,41], and miR-3129 [42]. EVs containing miR-21 induce the conversion of hepatic stellate cells (HSCs) into cancer-associated fibroblasts (CAFs), which secrete TGF-β, IL-6, IL-8, VEGF, and matrix metalloproteinases (MMP-2, MMP-9), thereby facilitating tumor progression, neoangiogenesis, EMT, and pre-metastatic niche formation. These EV subpopulations also act autocrinally on HCC cells, enhancing proliferation, migration, drug resistance, and the polarization of Kupffer cells toward the M2 phenotype, leading to suppression of anti-tumor immunity [40,41]. Several EVs also contribute directly to pre-metastatic niche formation. For example, the uptake of HCC-derived EVs containing miR-1247-3p by lung metastatic niche cells activates fibroblasts via β1-integrin/NF-κB signaling, priming the niche for metastasis [43]. Metastatic dissemination is further facilitated by EVs carrying lncRNAs such as MALAT1, which sponges miR-26a/b [44], and FAL1, which regulates ZEB1 and alpha-fetoprotein (AFP) by competing with tumor-suppressive miR-1236 [45]. Moreover, EVs carrying lncRNA-TUC339 interact with CAFs, promoting EMT, drug resistance, and metastatic dissemination, while they also induce M2 polarization in macrophages, impairing phagocytosis and promoting tumor progression. These EVs are among the most abundantly released in HCC [46]. On top of that, EVs containing vacuolar protein sorting-associated protein 4A (Vps4A) have a crucial role as tumor suppressors and regulators of exosome cargo sorting, preventing tumor-promoting exosomal cargo sorting (e.g., β-catenin). However, loss of its function leads to HCC progression and metastasis [47].

- **Tumor progression, migration, and drug resistance**

Meanwhile, EVs containing ATB sequester the miR-200 family and activate ZEB1/2, promoting drug resistance, migration, tumor progression, and metastasis [48]. Additionally, EVs carrying the lncRNA ROR [49] and miR-25 have also been shown to mediate resistance to sorafenib [50]. Other EVs containing circular RNAs (circRNAs) further drive HCC progression by sponging tumor-suppressive miRNAs [51,52,53,54,55], or EVs containing circRNAs with tumor-suppressive effects, such as circ-0051443, which sponges oncogenic miRNAs, can lead to HCC apoptosis [56]. EVs carrying miR-429 mediate the promotion of POU class 5 homeobox 1 (POU5F1) via targeting of Rb-binding protein 4 (RBBP4), leading to HCC progression [57]. HCC progression is also mediated by EVs carrying circ-0051443, which increases the survival of HCC cells and suppresses their apoptosis [58].

- **Suppression of anti-tumor immune responses**

EVs that carry miR-221 and miR-23a are some of the immunomodulatory EVs. The former cargo targets p27/Kip1, leading to its downregulation and leading to the impairment of cell cycle suppression and HCC cell proliferation, while it also induces disease progression by activating NFκB signaling [59,60]. EVs carrying miR-23a interact with NKs, preventing their activation and IFN-γ production, leading to immune evasion, while when they interact with macrophages, they lead to PD-L1 overexpression and T-cell immune response impairment [61]. In addition, the uptake of EVs containing let-7b by tumor macrophages (TAMs) induces cytokine release (e.g., IL-6) by the latter, promoting disease progression [62]. Additionally, EVs carrying PD-L1 suppress T-cell response (suppression of the immune checkpoint), leading to the tumor escape phenomenon [63], while those that carry TGF-β and circ-UHRF1 mediate T-cell exhaustion [64] and immune evasion by suppressing NK function via sponging of miR-449c-5p [65].

- **Neoangiogenesis and vascular permeability**

Several other EVs may induce modification in endothelial cells (ECs), aiming at increasing vascular permeability for tumor cells’ migration and metastasis. Some of these EVs carry miR-103, which modifies the expression of p120, ZO-1, and VE-cadherin in ECs, leading to increased permeability for enabling the intra- and extravasation of tumor cells [66]. Meanwhile, EVs that carry miR-210 crosstalk with ECs and downregulate SMAD4 and STAT6 expression in the latter under hypoxia, a phenomenon that enhances neoangiogenesis and metastatic dissemination [67]. In addition, it was shown that EVs containing H19 (lncRNA)–CD90(+) interact with human umbilical vein endothelial cells (HUVECs), inducing overexpression of VEGF and its receptor (VEGFR), leading to neoangiogenesis, as well as interact with hepatic stellate cells (HSCs), inducing fibrosis [68]. Additionally, the uptake of HCC-derived EVs carrying VEGF by ECs promotes angiogenesis and metastasis [69], while the reduction of EVs carrying CLEC3B (tumor suppressive cargo) or loss of its function lead to neoangiogenesis and distant metastasis [70].

Nevertheless, beyond hepatocytes, adipocytes, TAMs, MSCs, CAFs, and other TME cellular components also release EVs that have a crucial role in HCC progression [71,72,73,74,75,76,77,78,79,80,81,82,83,84,85,86,87,88,89]. In Table 1, we present some of those EVs of different origins and summarize several EV subpopulations involved in HCC pathogenesis [38,39,40,41,42,43,44,45,46,47,48,49,50,51,52,53,54,55,56,57,58,59,60,61,62,63,64,65,66,67,68,69,70,71,72,73,74,75,76,77,78,79,80,81,82,83,84,85,86,87,88,89].

### 3.2. Biliary Tract Cancer (BTC)

BTCs include different entities of tumors, based on their anatomical region, including (a) gallbladder cancer (GBC) and (b) cholangiocarcinoma (CCA), with the latter being subclassified into three distinct entities: (i) intrahepatic (iCCA); (ii) perihilar (pCCA), previously referred to as Klatskin; and (iii) distal (dCCA) CCA. ICCA is located in the bile ducts within the hepatic parenchyma, and dCCA in the bile ducts outside of the liver parenchyma and near the ampulla of Vater. At the same time, pCCA is at the junction of the right and left hepatic ducts. Meanwhile, there is another cancer entity called ampullary carcinoma, which is located at the site where the pancreatic duct empties into the second duodenal anatomical part. Additionally, there is also the mixed type CCA, presenting a phenotype similar to HCC and CCA [90,91].

#### 3.2.1. Gallbladder Cancer (GBC)

GBC has an age-standardized incidence of around 1.2 per 100,000 globally based on the latest data by GLOBACAN 2022, mostly identified in India and China, with approximately 89,045 deaths and ~122,469 cases in 2022. Several genetic aberrations have been identified in GBC cases, including loss of TP53, KRAS, and ERBB2/HER2, as well as PIK3CA mutation [92]. Several studies have demonstrated aberration in exosomal profiling in GC patients not only in comparison to healthy individuals, but also in those with benign pathologies such as cholecystitis. The study by Ueta, E. et al. demonstrated alterations (in silico) in the EV-miRNA signatures between patients with or without GC [93], while another in vitro study by Priya R et al. identified several proteins (approximately 268) that are embedded in GBC cell line-derived EVs, which are involved in GBC progression [94]. On top of that, Priya R et al. studied 86 EV proteins resulting from a quantitative proteomic analysis, which were at different levels in individuals with GBC in comparison to healthy controls. It resulted in three that could be potentially used for the detection of GBC in the early stage (29 out of 86), advanced stage (44 out of 86), or regardless of stage (13 out of 86) [95]. Another study by M. Kong et al. also demonstrated different EV profiling between GC patients, healthy individuals, and patients with benign pathologies such as cholecystitis. Moreover, a significant observation was made, as they identified alterations in exosomal membrane integrity with a notable reduction of unsaturated phosphatidylcholines and phosphatidylethanolamines in individuals with GC [96]. In Table 2, we demonstrate the key EVs that were identified in the aforementioned studies [94,95,96], and some other potentially serve as EV-based biomarkers from bile [97].

#### 3.2.2. Cholangiocarcinoma (CCA)

CCA constitutes a rare, highly aggressive GI malignancy accounting for 3% of total GI cancer cases. There is a gradually rising trend of iCCA incidence in Western countries (0.3–3.5 per 100,000); however, the rates remain higher for the Asian population (e.g., North-Eastern Thailand), a phenomenon that is mainly associated with the ingestion of raw fish that is infected with liver flukes, including Clonorchis sinensis and Opisthorchis viverrini [98]. Nevertheless, only 10% of total cases of primary hepatic cancers are attributed to iCCA, which constitutes the second cause after HCC. Meanwhile, there is a rare subtype, defined as the “mixed” type, which is a “hybrid” form of tumor consisting of transdifferentiated hepatocytes with a phenotype between CCA and HCC. On the other hand, dCCA and pCCA are more commonly diagnosed, with the former accounting for 20–30% of total cases, whereas the latter accounts for 50–60%. Unfortunately, due to the relatively asymptomatic course of this malignancy, diagnosis is usually in advanced stages [90,91,98,99]. Some other well-recognized risk factors for CCA development are MASLD, metabolic dysfunction, including obesity and T2DM, as well as underlying hepatobiliary diseases and malformations. PSC constitutes an autoimmune disease that affects biliary ducts, causing strictures and subsequently cholangiocarcinogenesis (22-fold higher risk for PSC patients, compared to non-PSC individuals) [90,100,101]. Additionally, chronic inflammation and cirrhosis caused by chronic viral hepatitis B and C infection, as well as choledocholithiasis, are strongly associated with CCA development. Some of the malformations that increase the risk of CCA development are Caroli disease and choledochal cysts. Meanwhile, CCA development is also attributed to nitrosamines found in ultra-processed foods, aflatoxin, as well as asbestos and thorotrast exposure [90,100,101,102]. Moreover, several mutations and epigenetic alterations have been identified in each distinct entity, with iCCA presenting mutations such as AR1D1A, IDH1/2, BAP1, and FGFR2 fusions. Meanwhile, dCCA and pCCA often present ERBB2 amplifications, as well as SMAD4, TP53, and KRAS mutations [90,101,103]. Despite the development of novel therapeutic and diagnostic approaches, CCA diagnosis remains challenging, as it is usually asymptomatic in advanced stages. The role of EVs in CCA is in the spotlight of current studies, considering their key role in the crosstalk between TME cellular components and CCA cells [104]. Several sources of EVs contribute to CCA progression. CCA-derived EVs carry oncogenic cargo such as miR-221, which promotes proliferation, survival, and PI3K/AKT activation [105,106], as well as miR-21, which activates the IL-6/STAT3 axis, suppresses PTEN and PDCD4, and drives EMT, invasiveness, and drug resistance. The latter subpopulation of EVs serves as a diagnostic biomarker in plasma and bile [106,107,108], while the downregulation of another cargo, such as miR-34c, facilitates CAF activation via WNT1 [109]. Other EV cargos include miR-30e, which suppresses EMT and limits dissemination [110]; miR-26a, which modulates KRT19 and β-catenin/GSK3β signaling [111]; the miR-200 family, which induces EMT and correlates with poor prognosis [112]; as well as miR-183-5p, which promotes angiogenesis, tumor progression, and chemoresistance through VEGF and PD-L1 pathways [113]. Circular RNAs such as circ-0000284 and circ-CCAC1 regulate LY6E and YY1 expression, enhancing malignant transformation, angiogenesis, and migration while serving as potential biomarkers [114,115]. Additional cargos include miR-192–5p, lncRNA-H19, EpCAM, ctDNA, MUC1, CLDN3, HER2, integrins, FZD10, vitronectin, lactadherin, BMI1, cytokines (TNF-α, IL-6), ceramides, LINC01812, and miR-210, all of which contribute to tumor growth, metastasis, EMT, immune evasion, fibrosis, or serve as diagnostic or prognostic markers [116,117,118,119,120,121,122,123,124,125,126,127,128].

Furthermore, EVs derived from the TME cellular components further influence CCA progression, such as TAM M2 EVs, which suppress CD8+T-cell cytotoxicity and promote angiogenesis through VEGF and circ-0020256 [129,130]; HSC EVs carrying miR-195 exerting tumor-suppressive effects [131]; as well as CAF-derived EVs containing miR-210, chemokines, and growth factors promoting EMT, tumor proliferation, and immune modulation [132,133]. Infection- and parasite-associated EVs also play key roles. HCV EVs, carrying viral proteins and RNA, drive inflammation, fibrosis, EMT, and oncogenic signaling [134], while HBV EVs (HBsAg, HBx, HBV-DNA) facilitate viral immune evasion, EMT, and neoplastic transformation [4,135,136]. Last but not least, dysbiotic microbiome-derived EVs activate TLR4/NF-κB signaling, promoting cholangiocarcinogenesis [137], while EVs from CCA cell lines (HuCCT1) and parasites (C. sinensis, O. viverrini) further contribute by modulating signaling pathways, promoting proliferation, invasion, angiogenesis, and immune evasion [138,139,140]. Table 3 summarizes these EV sources, cargos, and their roles in CCA pathogenesis.

In Table 3, we demonstrate some of the EVs that are involved in CCA pathogenesis.

### 3.3. Pancreatic Adenocarcinoma (PDAC)

PDAC remains a major global health burden, as it is highly malignant and one of the leading causes of cancer-related mortality worldwide. Its incidence and mortality rates continue to show a concerning upward trend, especially in Western European countries [141]. This phenomenon is attributed to obesity, high-fat diet, tobacco, and alcohol abuse, as well as chronic pancreatitis, type 2 Diabetes mellitus (T2DM), and family predisposition, including genetic hereditary syndromes. Interestingly, the oral microbiome plays a key role in PC development through bacterial translocation, which significantly increases the risk for pancreatic carcinogenesis, while many patients often present increased glucose levels or a T2DM diagnosis, even 3 years before PDAC primary diagnosis [142].

PDAC-derived EVs carry diverse cargoes that influence tumor progression, metastasis, and metabolic dysregulation. EVs containing KRAS, EGFR, CD44, or miR-222 correlate with tumor stage, progression, and poor prognosis [143,144,145,146]. EVs with miR-27a, CKAP4, or miR-125b-5p promote invasiveness, chemoresistance, and EMT via PI3K/Akt, MEK/ERK, or Wnt pathways [147,148,149,150,151,152,153], while miR-197-3p, miR-19a, miR-6796-3p, miR-4750-3p, miR-6763-5p, miR-450b-3p, miR-666-3p, miR-883b-5p, and miR-540-3p alter glucose metabolism, insulin secretion, or induce insulin resistance [145,150,151,152]. Moreover, EVs containing Caveolin-1, O-glycan-binding lectin, β2-microglobulin, Podocalyxin-like protein, S100A4, STAT14, LAMP1, Lin28B, Integrins, or MIF, as well as several other molecules modulate proliferation, apoptosis, metastatic dissemination, immune evasion, or stromal remodeling [145,154,155,156,157,158,159,160,161,162,163,164,165,166], while those EVs carrying Tspan8 enhance cell motility and migration [167]. EVs containing EphA2 serve as a predictive biomarker for therapy response [168], while EVs with CD151, CLDN4, EpCAM, LGALS3BP, MUC1, HIST2H2BE, or CLDN1 have an emerging role as a prognostic (worrisome prognosis), as they modify the tumor stroma and are associated with KRAS mutations and metastasis [144,169,170,171,172,173]. Pre-metastatic niche formation is further facilitated by EVs containing niche-related proteins or Annexin A6, metabolites, and miRNAs from CAFs (miR-21, miR-146, miR-155), PSCs (miR-21-5p, miR-451a, miR-5703, TGF-β-related proteins), and adipocytes, which provide metabolic support, promote EMT, ECM remodeling, and immune suppression [174,175,176,177,178,179,180,181]. Lastly, M2 macrophage-derived EVs carrying miR-155-5p or miR-221-5p enhance neoangiogenesis and tumor proliferation [182]. Collectively, these EVs contribute to PDAC pathogenesis [143,144,145,146,147,148,149,150,151,152,153,154,155,156,157,158,159,160,161,162,163,164,165,166,167,168,169,170,171,172,173,174,175,176,177,178,179,180,181,182] and are summarized in Table 4.

## 4. AI Applications in EV Research in Oncology

Given the high dimensionality and heterogeneity of EV cargo, AI methods are increasingly used to extract diagnostic, prognostic, and therapeutic signals from EV-derived multi-omics and biophysical profiles, with particular relevance to pancreatic and hepatobiliary malignancies, where early detection and treatment stratification remain challenging.

### 4.1. AI Methodologies Applicable to EV Research

- **ML, DL and multimodal models**

**Figure 2:**
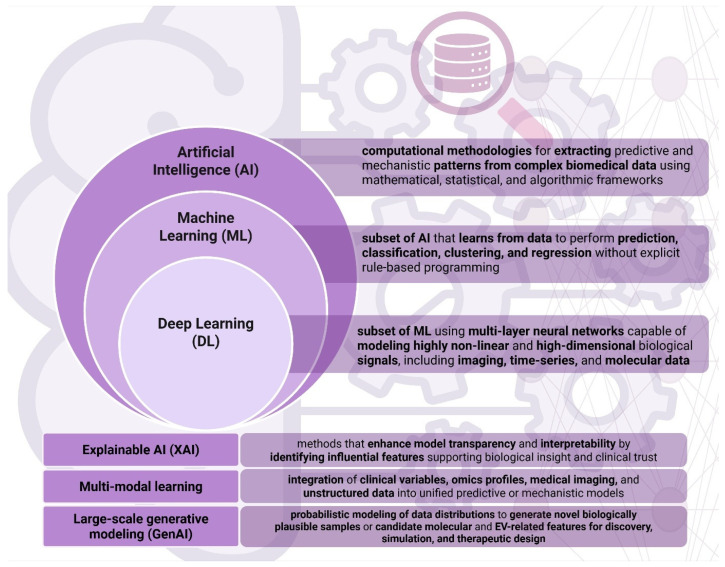
An overview of AI computational methodologies and implementation in EV-related research. Created in BioRender. Fortis, S. (2026) https://BioRender.com/uhb61dd (accessed 30 January 2026). Agreement license LC29AV9O1E.

Artificial intelligence (AI) and data science encompass computational methodologies designed to extract predictive and mechanistic patterns from complex biomedical data using mathematical, statistical, and algorithmic frameworks [183,184,185]. In biomedical applications, these pipelines typically include preprocessing, feature engineering, model training, evaluation, and deployment with monitoring [186]. Within this framework, machine learning (ML) methods enable supervised and unsupervised prediction, classification, and clustering across heterogeneous datasets [184,187], while deep learning (DL) extends ML by employing multilayer neural networks capable of modeling highly non-linear and high-dimensional signals, including imaging, time-series, and molecular data [188,189,190]. Recent advances further incorporate multimodal learning architectures that integrate clinical variables, omics profiles, medical imaging, and unstructured data into unified predictive models [191,192], large-scale generative modeling for representation learning and data augmentation [193], and explainable artificial intelligence (XAI) approaches that enhance transparency and biological interpretability [194,195]. Collectively, these computational strategies are increasingly applied to extracellular vesicle (EV) research to support biomarker discovery, signal extraction, and outcome prediction in cancer and metabolic disease [196,197,198,199,200]. Figure 2. Provides qn overview of AI computational methodologies and implementation in EV-related research, created in BioRender. Fortis, S. (2026) https://BioRender.com/uhb61dd (accessed 30 January 2026).

- **Pattern discovery and non-linear modeling**

A principal advantage of AI-driven modeling lies in its ability to capture complex, non-linear, and high-dimensional biological relationships that are not adequately represented by traditional linear statistical methods [183,188]. This capability is particularly relevant in EV research, where tumor evolution, intercellular communication, immune modulation, and molecular signaling are governed by multi-scale, non-linear interactions across cellular and molecular layers [196,201,202,203,204]. EV-mediated processes reflect dynamic biological systems in which cargo composition, cellular origin, and microenvironmental context interact in non-additive ways, rendering AI-based approaches well-suited for extracting disease-relevant patterns from EV-derived data.

- **AI in EV-based biomarker identification**

Advanced computational approaches, including ML, DL, and multimodal architectures, enable the integration of neural networks, gradient-boosting models (e.g., CatBoost), and representation-learning frameworks to detect subtle yet biologically meaningful EV signatures across imaging, proteomic, transcriptomic, and clinical data modalities [188,189,190,191,192,193,205]. These methods uncover disease-specific biomarker patterns that support early cancer detection, patient stratification, treatment-response prediction, and precision oncology, revealing EV-driven biological signals that are systematically missed by linear statistical models [190,201,203,206]. ML algorithms learn predictive non-linear relationships directly from data, whereas DL models capture complex EV distributions and tumor–microenvironment interactions, thereby enabling the discovery of EV-based biomarkers and mechanistic insights that are inaccessible to conventional statistical approaches [202,203,204,207,208,209,210]. Importantly, growing empirical evidence demonstrates that EV biomarkers identified using AI-based methodologies cannot be recovered using traditional statistical frameworks, underscoring the necessity of modern computational approaches for EV research [199,200,206]. In a study on metabolic dysfunction-associated steatotic liver disease (MASLD), Trifylli et al. (2025) applied explainable gradient-boosting models to plasma-derived EV lipidomic and proteomic profiles and identified non-linear lipid–protein interaction patterns predictive of histologic steatosis stage [206]. Classical univariate and multivariate statistical analyses failed to detect these associations, whereas SHAP-based feature attribution revealed biologically coherent EV signatures [194,195,206]. A complementary analysis by the same group further demonstrated that EV-derived biomarkers exhibit complex, non-monotonic behavior and interaction effects that cannot be adequately modeled using linear regression or correlation-based approaches [207]. Together, these studies provide direct evidence that AI enables biomarker discovery in EV research precisely because EV-associated biological signals follow high-dimensional, non-linear distributions, highlighting the fundamental limitations of traditional statistical methods and the essential role of ML and DL in modern EV-based precision diagnostics [210,211,212,213,214,215,216,217,218].

### 4.2. AI-Enhanced EV-Based Diagnosis, Prognosis, and Prediction in Oncology

- **AI-enhanced SERS for EV classification**

AI-enhanced analysis of extracellular vesicles using label-free Raman and surface-enhanced Raman spectroscopy (SERS) has emerged as a promising approach for cancer detection and EV phenotyping. Although many studies span multiple tumor types, the methodological principles are directly applicable to EV-based diagnostics and prognosis in pancreatic and hepatobiliary cancers. By learning complex, non-linear spectral signatures reflecting EV biochemical composition, ML and DL models can discriminate malignant from non-malignant EV populations, identify tissue-of-origin patterns, and support minimally invasive diagnostics [208].

One of the most advanced studies in this domain is the work of Shin et al. (2023), who analyzed plasma-derived exosomes from six cancer types using label-free SERS coupled with a deep-learning classifier. In a large cohort including 520 independent test samples, their Exosome-SERS-AI system achieved high diagnostic performance for cancer detection (AUC ≈ 0.97) and tissue-of-origin prediction (mean AUC ≈ 0.95), demonstrating the feasibility of clinically meaningful SERS-EV diagnostics supported by rigorous patient-level data splitting and independent test evaluation [209]. Complementing this, Liu et al. (2024) provided a comprehensive review of label-free SERS-based exosome analysis for cancer diagnosis, summarizing studies that apply linear and non-linear ML methods, including PCA-LDA, PLS-DA, SVM, and CNNs, across lung, breast, hepatocellular, head and neck, and other cancers, with several reports achieving classification accuracies above 90% under controlled experimental conditions [210]. In breast cancer, Xie et al. (2022) further demonstrated that AI-assisted analysis of serum exosome SERS profiles can distinguish malignant from benign disease and detect post-operative residual disease, supporting the potential of SERS-EV platforms for longitudinal monitoring [211].

By contrast, the recent study by del Real Mata et al. (2025) represents an earlier-stage methodological exploration rather than a clinically validated diagnostic framework. The authors developed a single-EV SERS spectral library from glioblastoma and medulloblastoma samples and compared linear, tree-based, and convolutional models across multiple preprocessing and SHAP-based feature-selection pipelines. Although internal accuracies of approximately 83% (multi-cell-line) and 91% (binary cancer vs. non-cancer) were reported, the study is limited by a very small human cohort (10 cancer, 10 control), spectrum-level rather than patient-level splitting, potential information leakage during preprocessing and feature selection, and reliance on accuracy without reporting AUC or external validation. Consequently, this work should be interpreted as a proof-of-concept rather than evidence of clinically deployable EV-based AI diagnostics [212].

Collectively, high-quality SERS-EV studies incorporating robust patient-level validation (e.g., Shin et al.) [209] and systematic methodological appraisals (e.g., Liu et al.) [210] demonstrate that AI applied to Raman spectral EV profiling can accurately classify malignancies and support early cancer detection. At the same time, earlier methodological studies highlight the necessity of rigorous data partitioning, standardized spectral preprocessing pipelines, and independent cohort validation to ensure translational reliability.

- **AI in microflow cytometry and EVMAP**

Beyond conventional classification tasks, ML has demonstrated value in cancer risk prediction by extracting high-dimensional features from extracellular vesicle (EV) cytometry data. A representative example is the work by Paproski et al. (2023), who developed the Extracellular Vesicle Machine Learning Analysis Platform (EVMAP) to analyze microscale flow cytometry (lFCM) data from plasma EVs, particularly PSMA- and ghrelin-positive vesicles, combined with clinical variables for prediction of high-grade (grade group ≥ 3) prostate cancer in men referred for biopsy [213]. Using patient-level five-fold cross-validation on a 215-patient cohort, their optimized XGBoost-based model generated continuous disease-risk scores and significantly outperformed manual gating, achieving an ROC–AUC of 0.75 compared with 0.52 for conventional EV gating.

These findings illustrate how AI-driven analysis of EV microflow cytometry can capture subtle, clinically relevant phenotypic patterns that are not apparent with human-interpretable gating strategies, highlighting the potential of such platforms to improve risk stratification. However, the reported performance relies solely on internal cross-validation without replication in independent cohorts, and the generalizability of the model remains to be established in larger, multicenter studies [213].

## 5. AI Applications in EV Research in Pancreatic and Hepatobiliary Malignancies

### 5.1. EV-Multi-Omics in GI Malignancies

ML-based EV proteomic analyses have shown considerable promise for identifying tumor-specific biological signals relevant to GI malignancies. A meta-analysis by Bukva et al. (2023) demonstrated that EV-derived protein profiles from 60 cancer cell lines contain distinct tumor-type-specific signatures and can predict functional phenotypes, such as invasion capacity and proliferation rate, using logistic regression and LASSO-regularized models. Notably, selected EV protein panels achieved classification accuracies of up to 96.6% across nine tumor types, while regression models yielded R^2^ values of 0.68 for invasion and 0.62 for proliferation [214].

These results support the potential of EV-based multi-omics and ML frameworks for refining cancer stratification and pathway-level interpretation. However, the analysis relies entirely on in vitro EVs released from cancer cell lines, with a limited sample size and no patient-derived data or external validation, which substantially limits clinical generalizability. Consequently, while these findings provide valuable biological insight, they require confirmation in plasma-derived EV datasets from well-characterized GI cohorts [214].

### 5.2. HCC Surveillance Models

AI has demonstrated substantial potential in improving HCC surveillance and preclinical detection in high-risk populations. In a large Asian cohort, Kwok et al. developed an ML model based on routine blood-test parameters for early HCC detection. Trained on 3415 HCC cases, the algorithm outperformed ultrasound plus AFP screening (43.7% sensitivity), achieving 79.4% sensitivity for detection 1–30 days before clinical diagnosis while maintaining specificity above 75% across all evaluated windows [215].

Long-term HCC risk prediction has also benefited from AI-based methodologies. Minami et al. (2023) developed and externally validated the SMART model, a seven-parameter random survival forest algorithm for stratifying HCC risk after sustained virologic response to hepatitis C therapy. Using derivation (n = 1742) and external validation (n = 977) cohorts, the model achieved a c-index of 0.839, outperforming established scores such as aMAP and ADRES and enabling individualized five-year risk estimation [216]. In chronic hepatitis B, Angelakis et al. (2024) conducted a multicenter evaluation of the PAGE-B score and assessed whether ML approaches could enhance HCC risk prediction. A conditional survival forest model integrating PAGE-B variables with cirrhosis status achieved concordance values of 0.86 in training and 0.85 in test cohorts, outperforming Cox proportional hazards models based on PAGE-B variables alone (0.79–0.80) [217]. Collectively, these results indicate that non-linear ML-based survival modeling may provide superior risk discrimination compared with traditional statistical approaches.

Nevertheless, most available studies rely on retrospective cohorts enriched with high-risk individuals, raising concerns regarding spectrum bias and real-world generalizability. The short pre-diagnostic prediction window in Kwok et al. may inflate performance estimates, while etiologic and geographic diversity remain limited in the SMART and CSFM cohorts. Prospective, multi-ethnic validation and formal assessment of clinical utility will be required before widespread implementation [215,216,217].

AI has also been applied to the biophysical profiling of EVs for cancer detection using label-free vibrational spectroscopy. Uthamacumaran et al. evaluated Raman and FTIR spectra of serum-derived EVs from patients with hepatocellular, pancreatic, colorectal, and breast cancer (n = 9) and healthy controls (n = 5). After baseline correction, multiple classical ML algorithms, including AdaBoost-Random Forests, Decision Trees, and Support Vector Machines, were trained to distinguish cancer-derived from healthy EV spectra. Using a random 50:50 train–test split, apparent diagnostic accuracies exceeding 90% were reported for Raman-based classification, with FTIR achieving approximately 80% accuracy [218]. However, this study should be interpreted strictly as exploratory. The sample size was extremely small, the analysis was performed at the spectrum level rather than the patient level, the random split likely induced information leakage, and no external or temporally independent validation was performed. These factors substantially inflate reported performance and preclude clinical inference [218].

### 5.3. PDAC Diagnosis and Prediction

In addition to AI-enhanced EV profiling via Raman and FTIR spectra, there are some other AI-driven methods that have substantially advanced non-invasive biomarker discovery for PDAC, particularly through extracellular vesicle molecular profiling and integration with radiologic data. Cyst-X, an MRI-based deep-learning framework, demonstrated clinically meaningful performance for predicting malignant transformation in intraductal papillary mucinous neoplasms. Using multicenter MRI data from 764 patients across seven institutions, the classifier achieved an AUC of 0.82, outperforming Kyoto guidelines and expert radiologists and demonstrating robust generalization in both centralized and federated learning settings [219]. Limitations include reliance on retrospective data, absence of biochemical or EV integration, and lack of prospective validation.

Small EV transcriptomics has also emerged as a promising PDAC liquid-biopsy modality. Liu et al. (2025) analyzed plasma sEV mRNA profiles from 100 individuals using four supervised ML feature-selection approaches. A four-mRNA diagnostic signature combined with CA19-9 achieved AUCs of 0.902–0.971 in training and 0.803–0.938 in validation, and the derived risk score remained an independent prognostic factor in Cox regression. Limitations include modest sample size, single-center recruitment, risk of overfitting, and lack of external multicenter validation [220].

Large-scale EV transcriptomic profiling was further extended by Liang et al., who analyzed 852 EV transcriptomes totaling 6.75 Tbp of sequencing data across two cohorts. Recursive feature elimination identified 31 transposable element-associated EV RNAs, and an SVM classifier achieved AUCs of 0.90 (training), 0.86 (test), and 0.88 (external validation), supporting reproducibility across independent datasets [221]. Remaining limitations include the need for mechanistic validation of transposable-element biology in EVs and reliance on bulk EV isolates, which may obscure cell-type specificity.

**Figure 3:**
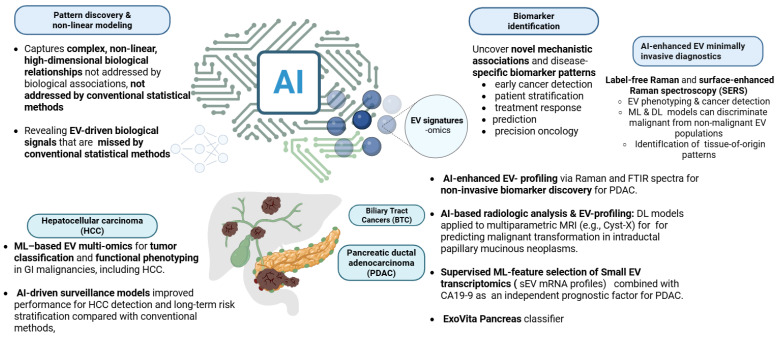
AI applications in EV research in pancreatic and hepatobiliary malignancies. Created in BioRender. Fortis, S. (2026) https://BioRender.com/wg293c9 (accessed on 30 January 2026). Agreement license: YG29AVAF5W.

Proteomic EV biomarkers have also shown strong diagnostic potential. Hinestrosa et al. developed the ExoVita Pancreas classifier using plasma EV proteins from 650 individuals, including 105 stage I/II PDAC cases. Using two feature-selection schemes and rigorous cross-validation, the model achieved an AUC of 0.971 (93.3% sensitivity, 91.0% specificity) and maintained high performance in an external validation cohort (n = 113). Remaining challenges include the absence of head-to-head comparisons with clinically used risk scores and the need for standardized EV isolation pipelines for real-world deployment [222]. Notably, EV-focused ML studies in biliary tract cancers remain extremely limited. Given the rising incidence and poor prognosis of these tumors, dedicated AI-EV multi-omics research in this domain represents an important unmet clinical and computational need [222]. Figure 3. Demonstrates some of the key AI applications in EV research in pancreatic and hepatobiliary malignancies, created in BioRender. Fortis, S. (2026) https://BioRender.com/wg293c9. YG29AVAF5W (accessed on 30 January 2026). 

## 6. AI-Assisted EV Therapeutic Engineering and Drug Discovery

AI is increasingly transforming EV-based therapeutic engineering and drug discovery. Modern computational approaches, including supervised ML, DL, graph neural networks (GNNs), and generative models, enable systematic interrogation of ligand–receptor interactions, prediction of EV-cell tropism, optimization of EV cargo loading, and large-scale virtual screening for EV-mimetic lipid nanoparticles. These methods accelerate hypothesis generation and rational therapeutic design, substantially reducing the time required to progress from preclinical discovery to translational development [223].

### 6.1. EV Databases

AI-driven EV research relies heavily on curated multi-omics databases, including Vesiclepedia, ExoCarta, EV-TRACK, ExoRBase, EVmiRNA, and EVpedia-Lipid. These repositories integrate transcriptomic, proteomic, lipidomic, and experimental metadata from thousands of EV studies. When combined with modern ML and AI workflows, these datasets enable high-throughput biomarker mining, ligand–receptor prediction, EV subtype characterization, and in silico identification of candidate therapeutic cargoes, thereby providing foundational infrastructure for data-driven EV engineering [224].

### 6.2. AI-Optimized EV Cargo Design and Ligand/Target Prediction Models

AI is increasingly incorporated into the design, optimization, and evaluation of EV therapeutics. EVs offer inherent advantages as delivery vehicles, including biocompatibility, immune evasion, and selective cellular tropism, and recent advances in computational modeling have accelerated progress in EV engineering. Lu et al. (2025) introduced an interpretable graph-based machine-learning framework for predicting ligand–receptor interactions governing EV-cell targeting. By integrating multi-omics data, chemical structure descriptors, and receptor–ligand affinity features, the model identified ligand–receptor pairs associated with EV uptake and therapeutic response, enabling in silico simulation of EV-cell communication dynamics and supporting rational design of targeted EV therapeutics, particularly for EV-delivered miRNA and protein payloads [225]. Complementing these biological interaction models, Wang et al. (2024) applied AI-assisted virtual lipid screening to the large-scale design of ionizable lipids for artificial vesicles. Their pipeline evaluated approximately 20 million candidate lipids using neural-network prediction of pKa values and mRNA-delivery probability, yielding experimentally validated synthetic vesicles with enhanced structural stability and intracellular delivery efficiency. Although these vesicles are not natural EVs, the computational strategies are directly applicable to hybrid EV–nanoparticle systems and rational lipid engineering for EV-mimetic delivery platforms [226]. In parallel, Kumar et al. (2024) provided comprehensive evidence that EV-encapsulated RNAs and proteins can be optimized as therapeutic cargos in cancer, metabolic disease, and immune disorders. While this work is a review rather than an AI-driven modeling study, it establishes the biological rationale supporting the development of AI-guided cargo selection frameworks [227]. Despite strong progress, current AI-based EV-engineering studies face notable constraints. Most rely on incomplete ligand–receptor annotations, limited training datasets, and predominantly in vitro validation. AI-designed lipid formulations often lack systematic in vivo pharmacokinetic and immunogenicity assessment, and no existing model fully integrates EV heterogeneity, biodistribution, or dynamic tissue microenvironments. Larger multi-omics datasets, systematic benchmarking, and unified validation frameworks will therefore be required to enable reliable clinical translation of AI-engineered EV therapeutics.

### 6.3. AI-Guided Precision Therapy and EV-Based Drug Resistance

AI is increasingly incorporated into precision oncology to improve individualized treatment selection, particularly for hepatobiliary and pancreatic malignancies. By integrating clinical variables, genomic alterations, epigenetic dysregulation, prior treatment history, drug–drug interaction profiles, and toxicity risk, AI-based models can generate patient-specific therapeutic strategies that extend beyond conventional guideline-based decision making. EV-derived molecular signatures further strengthen this paradigm. Multiple studies have demonstrated that EV-associated miRNAs can predict acquired resistance to systemic therapies such as sorafenib in hepatocellular carcinoma and gemcitabine in pancreatic ductal adenocarcinoma, establishing EV-based liquid biopsy as a minimally invasive tool for treatment selection and real-time monitoring of therapeutic response [228]. Despite this promise, most EV-based resistance studies remain retrospective and exploratory, with limited cohort sizes and heterogeneous EV isolation and profiling protocols. The extent to which EV-derived resistance signatures generalize across etiologies, treatment regimens, and patient populations remains uncertain. Prospective validation and standardized EV-processing pipelines will therefore be essential before such AI-driven resistance-prediction frameworks can be incorporated into routine clinical decision making.

**Figure 4:**
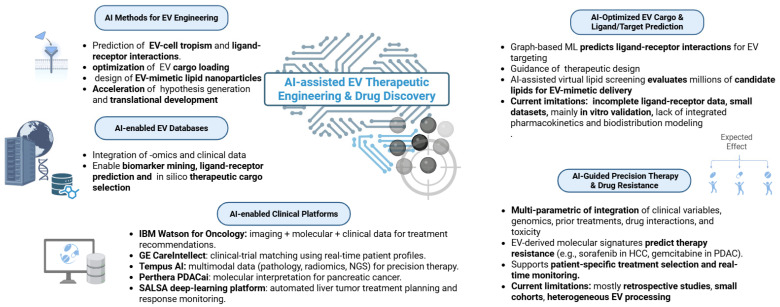
AI-assisted EV therapeutic engineering and drug discovery. Created in BioRender. Fortis, S. (2026) https://BioRender.com/mhdak8i (accessed on 30 January 2026). Agreement license: UU29AVAXB8.

AI-enabled computational decision-support systems are becoming increasingly prominent in clinical oncology. IBM Watson for Oncology integrates imaging features, molecular data, and structured clinical parameters to recommend evidence-based treatment options. GE HealthCare’s CareIntellect for Oncology supports clinical-trial matching by analyzing real-time patient profiles. Tempus AI combines multimodal inputs, including pathology images, radiomics, next-generation sequencing, and transcriptomics, to guide precision treatment. Perthera’s PDACai applies AI-driven molecular interpretation to support therapeutic recommendations in pancreatic cancer. In hepatobiliary malignancies, the SALSA deep-learning platform developed by the Vall d’Hebron Institute of Oncology enables automated treatment planning and response monitoring for liver tumors, illustrating how AI-assisted radiology and clinical modeling can streamline oncologic workflows [229,230,231,232,233]. While these platforms illustrate the clinical feasibility of AI-assisted oncology, their integration with EV-derived biomarkers remains limited. Most systems rely primarily on imaging and tissue-based molecular profiling rather than circulating EV data. Future iterations of clinical decision-support tools could benefit from incorporating EV-based liquid-biopsy features to enable longitudinal treatment monitoring and adaptive therapy selection. However, regulatory approval, interpretability, and prospective evaluation in real-world oncology pathways will be required before EV-integrated AI systems can achieve widespread clinical adoption. Figure 4. AI-assisted EV therapeutic engineering and drug discovery. Created in BioRender. Fortis, S. (2026) https://BioRender.com/mhdak8i (accessed on 30 January 2026).

## 7. Challenges for AI in EV-Based Oncology Research

Despite rapid methodological progress, the integration of artificial intelligence with EV-based precision oncology remains technically demanding and requires careful experimental and computational design. EV-derived biomarker studies are characterized by substantial biological and technical heterogeneity, and many AI models are trained on relatively small or single-center cohorts. These characteristics do not preclude meaningful modeling; rather, they necessitate expert-level methodological practice, including rigorous validation strategies, biologically informed model design, and appropriate statistical learning frameworks.

Importantly, many reported “limitations” in EV–AI studies arise not from intrinsic constraints of AI or EV biology, but from suboptimal experimental design, improper validation, or inappropriate model selection. Differences in EV isolation, molecular profiling, and preprocessing can introduce technical variability that may be misinterpreted by AI systems as biological signal if not handled with domain-aware computational strategies. Consequently, transparent reporting, reproducible analytical pipelines, and prospective evaluation are essential to ensure safety, interpretability, and real-world clinical utility. Addressing these challenges is therefore not a limitation of AI per se, but a prerequisite for its responsible deployment in EV-informed precision oncology [234].

### 7.1. EV Standardization and Reproducibility

- **Data quality, standardization, and reproducibility**

EV research is intrinsically subject to variability in isolation protocols, quantification methods, and annotation standards. Heterogeneous sample-preparation pipelines, variable particle purity, and platform-dependent measurement biases introduce batch effects that directly affect model behavior. EVs can be isolated from cultured cells and diverse biological fluids, with differential centrifugation being the most commonly used method. Large EVs (microvesicles/oncosomes) are isolated at lower centrifugal forces (10,000–20,000× *g*), whereas exosomes require higher forces (~100,000× *g*) via ultracentrifugation, which frequently results in co-isolation of non-vesicular particles such as lipoproteins and protein aggregates [235,236,237,238]. According to the MISEV2023 guidelines, no currently available isolation strategy achieves complete removal of non-vesicular particles, underscoring the intrinsic complexity of EV preparations [32].

Combining multiple isolation approaches can improve EV purity but increases experimental complexity and processing time. EV marker heterogeneity further complicates interpretation, particularly in patients with multimorbidity or overlapping inflammatory and oncologic processes [239]. Storage-related factors also influence EV integrity; repeated freeze–thaw cycles may disrupt vesicles and alter cargo composition, especially for RNA species. Although storage at −80 °C is considered optimal, alternative stabilization strategies such as freeze-drying with cryoprotectants have been proposed. Manufacturing challenges, including production cost and yield limitations, and incomplete understanding of EV uptake and biodistribution further hinder translation [240,241].

Without appropriate experimental control and computational awareness, such technical artifacts may be learned by AI systems as predictive features, leading to spurious biomarker discovery and inflated performance estimates. The MISEV2023 guidelines therefore recommend inclusion of EV-depleted controls, comprehensive methodological disclosure, and multiparametric EV characterization to ensure transparency and reproducibility [32]. Harmonized EV frameworks and standardized reporting remain essential prerequisites for clinically meaningful AI development.

**Figure 5:**
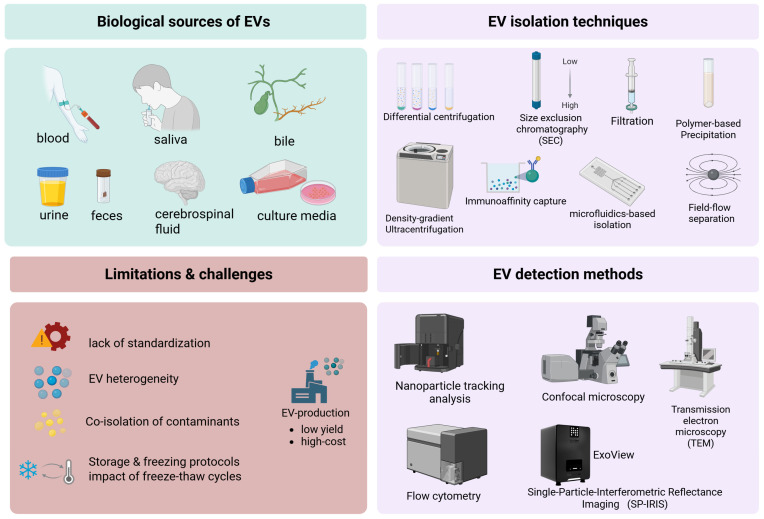
EV isolation and detection methods—limitations of EV utilization. EVs can be isolated from cultured cells and diverse biological fluids, with differential centrifugation being the most commonly used method, where large EVs are isolated at lower centrifugal forces and exosomes at higher forces via ultracentrifugation. Alternative approaches include size exclusion chromatography, density gradient ultracentrifugation, polymer-based precipitation, immunoaffinity capture, microfluidics-based isolation, and ultrafiltration, each with distinct advantages and limitations. EV detection and characterization rely on flow cytometry, nanoparticle tracking analysis, and imaging techniques, while EV heterogeneity, lack of standardization, storage-related integrity issues, and limited understanding of EV uptake and biodistribution remain major challenges for clinical translation [32,235,236,237,238,239,240,241,242]. Created in BioRender. Fortis, S. (2026) https://BioRender.com/wchnzdy (accessed on 25 January 2026). Agreement license: MC29A7O269.

Alternative isolation strategies include size-exclusion chromatography, density-gradient ultracentrifugation, polymer-based precipitation, immunoaffinity capture, microfluidics-based isolation, and ultrafiltration, each with distinct advantages and limitations. EV detection and characterization rely on flow cytometry, nanoparticle tracking analysis, and imaging techniques. Nevertheless, EV heterogeneity, lack of standardization, storage-related integrity issues, and limited understanding of EV uptake and biodistribution remain major obstacles to clinical translation. In Table 5, we summarize the advantages and limitations of common EV isolation and detection approaches [235,236,237,238,239,240,241,242], while in Figure 5. EV isolation and detection methods—limitations of EV utilization, created in BioRender. Fortis, S. (2026) https://BioRender.com/wchnzdy (accessed on 25 January 2026). Agreement license: MC29A7O269.

- **Limited labeled datasets and cohort diversity**

Many EV–AI studies rely on relatively small, single-center cohorts due to biospecimen constraints and the cost of multi-omics profiling. This leads to high feature-to-sample ratios and increases susceptibility to overfitting and population bias. However, these challenges are not unique to EV research and can be mitigated through appropriate methodological design, including nested cross-validation, external or temporal validation, regularized modeling, and feature-stability analysis. Expansion of multi-institutional EV biobanks, adoption of federated learning frameworks, and responsible synthetic data augmentation will further enable scalable EV–AI modeling while preserving privacy and improving generalizability.

Crucially, these issues do not indicate that AI is unsuitable for EV-based oncology research; rather, they highlight the necessity for expert methodological leadership. Addressing small sample sizes, protocol heterogeneity, and multimodal data integration requires advanced statistical learning strategies, representation learning, and explainable modeling frameworks. Meaningful application of AI in EV research therefore depends critically on the involvement of experienced AI and data science specialists who can ensure correct model formulation, leakage-safe validation, and biologically coherent interpretation, thereby preventing spurious associations and maximizing translational relevance.

- **Model interpretability and biological explainability**

Although deep learning excels at feature extraction, black-box models limit mechanistic interpretation, clinician trust, and regulatory acceptance. In EV-based biomarker research, interpretability is particularly critical for distinguishing true biological drivers from protocol-dependent artifacts. Explainable AI (XAI) methods are therefore indispensable for identifying influential EV features, supporting hypothesis generation and enabling clinically actionable insight. Integration of XAI into EV–AI workflows also facilitates reproducibility and regulatory auditability [206,207].

- **Integration of multimodal EV-omics and clinical data**

EV-derived proteomics, lipidomics, nucleic acid profiling, and imaging data are frequently analyzed in isolation despite representing complementary aspects of tumor biology. Fragmented modeling reduces robustness and biological interpretability. Cross-modality fusion architectures, self-supervised representation learning, and graph-based biomedical knowledge networks provide promising strategies for unifying EV-omics with imaging, clinical records, and longitudinal trajectories, enabling more faithful modeling of tumor evolution and treatment response.

These requirements reinforce that EV-based AI oncology research must be conducted within genuinely interdisciplinary teams combining EV biology, clinical oncology, and advanced AI methodology.

### 7.2. Clinical, Ethical, and Regulatory Considerations

- **Clinical validation and prospective trials**
  Most EV–AI studies remain retrospective. Translation into routine clinical workflows requires rigorous prospective validation in real-world cohorts and longitudinal surveillance settings. Such trials must assess not only predictive performance but also clinical utility, decision impact, and cost-effectiveness within established diagnostic and therapeutic pathways.
- **Regulatory pathways and safety frameworks**
  EV–AI platforms intended for diagnostic or therapeutic decision support must comply with evolving digital health regulatory standards, including requirements for algorithmic transparency, robustness testing, and post-deployment performance monitoring. For AI-guided EV engineering, additional constraints apply, such as compatibility with Good Manufacturing Practice (GMP), biosafety evaluation, and quality control of engineered vesicle products. Early engagement with regulatory authorities and incorporation of auditability and traceability into model design are therefore critical for successful clinical translation [243].
- **Equity, fairness, and global generalization**
  Disparities in cohort composition and data availability may propagate algorithmic bias and disproportionately affect underrepresented populations. EV–AI models trained on geographically or etiologically restricted datasets may perform poorly when deployed in heterogeneous healthcare environments. Fairness-aware learning strategies, balanced cohort acquisition, and international benchmarking initiatives are thus essential to ensure equitable deployment of EV–AI tools across diverse patient populations [243]. Table 6 summarizes the principal biological, technical, and translational challenges associated with the implementation of AI in EV-based oncology research.

Taken together, the challenges outlined above reflect intrinsic biological complexity and translational constraints rather than inherent limitations of artificial intelligence itself. As summarized in Table 6, many commonly cited “limitations” arise from experimental heterogeneity, incomplete biological knowledge, and insufficient methodological rigor rather than from fundamental barriers to AI modeling. When EV-based oncology research is conducted within interdisciplinary teams that combine EV biology, clinical oncology, and advanced AI expertise, these challenges can be systematically identified, controlled, and transformed into solvable methodological problems. Accordingly, the reliable clinical translation of EV–AI systems depends not only on data availability and experimental standardization, but critically on expert-level AI design, validation strategies, and biologically informed model interpretation, ensuring that learned signals reflect true disease biology rather than protocol-dependent artifacts.

## 8. Emerging AI-Assisted EV Research Methodologies and Future Perspectives

Building on the methodological and translational challenges outlined in Section 7, several emerging AI-assisted EV methodologies aim to address these limitations through advances in imaging, spectroscopy, and multimodal learning.

### Computer Vision EV Analysis

Computer vision and ML have increasingly been applied to automate the characterization of EVs, reducing the subjectivity, labor intensity, and limited throughput inherent to manual imaging workflows. Transmission electron microscopy (TEM) remains one of the most widely used visualization techniques for EVs, but manual particle identification is time-consuming and operator-dependent. Kotrbová et al. developed one of the earliest semi-automated software tools for TEM-based EV analysis, enabling standardized identification of EV-like structures across staining protocols and improving reproducibility compared with fully manual assessment. However, their approach relied on handcrafted image-processing rules rather than modern ML-based classification, limiting scalability and broader applicability [244].

ML-enabled computer-vision approaches have also been explored for NTA and microscopy-based quality control. Xu et al. demonstrated how supervised ML and image-analysis algorithms can be applied to NTA data to improve EV size-distribution estimation, correct instrument artefacts, and reduce false-positive particle detection, thereby supporting scalable EV characterization workflows [245]. More advanced image-based pipelines have been introduced for multimodal chemical imaging. Bamford et al. combined time-of-flight secondary ion mass spectrometry (ToF-SIMS) with an unsupervised self-organizing map/relational perspective mapping (SOM-RPM) algorithm to perform high-resolution chemical imaging of microglia-derived EVs, enabling pixel-level discrimination of EVs from control versus lipopolysaccharide-stimulated cells and revealing neuroinflammation-associated biochemical alterations such as reduced cysteine thiol content [246]. Similarly, advances in nano-imaging have incorporated ML to refine single-particle EV detection. Xu et al. compared nanoflow cytometry and nanoimaging modalities and applied ML-based correction models to reduce false-positive particle identification, improving the reliability of single-vesicle quantification [245].

Although these studies demonstrate promising methodologies, they remain largely exploratory. Most rely on small experimental datasets, often restricted to cell-line-derived EVs, use internal cross-validation without independent replication, and frequently apply ML post hoc to pre-extracted features rather than fully end-to-end learning pipelines. Standardization across imaging platforms, staining procedures, and sample-preparation protocols remain limited, and none of the existing tools has yet been systematically evaluated in clinical EV cohorts. Furthermore, integration of EV-omics with imaging, pathology, and electronic health records remains incomplete in routine clinical settings. Prospective, multicenter studies, aligned with standardized EV characterization guidelines and transparent AI development practices, are needed to confirm diagnostic accuracy, clinical utility, and patient benefit. Addressing these challenges will be essential to enable the reliable integration of EV–AI platforms into oncology diagnostics, surveillance, and therapeutic decision-making, ultimately supporting safe and effective deployment in real-world precision cancer care.

Next-generation EV–AI innovations are expected to incorporate multimodal foundation models trained on EV-omics, radiomics, and clinical corpora, self-supervised and transfer-learning pipelines to reduce dependence on large labeled datasets, and real-time digital EV biomarkers to monitor therapeutic response and resistance evolution. Generative AI approaches may accelerate rational design of EV cargo and membrane engineering, while federated and privacy-preserving learning systems will enable collaborative model training across international EV biobanks. Clinically integrated EV–AI decision-support systems hold promise for adaptive precision oncology workflows. The convergence of EV biology, artificial intelligence, computational drug design, and precision oncology offers a pathway toward earlier cancer detection, dynamic treatment adaptation, and biologically informed therapeutics. Continued investment in data standardization, ethically aligned AI frameworks, prospective clinical evaluation, and interdisciplinary collaboration will be essential to unlock the full translational impact of EV–AI systems [247,248].

## 9. Conclusions

EVs play a pivotal role in cancer biology as mediators of intercellular communication, tumor progression, immune modulation, and metastatic dissemination. Their molecular cargo provides a minimally invasive window into tumor dynamics, enabling early cancer detection, risk stratification, therapeutic monitoring, and identification of actionable molecular targets. Advances in AI have further accelerated EV research by enabling large-scale multi-omics integration, modeling of non-linear biological interactions, and rapid discovery of candidate biomarkers and engineered EV-based therapeutic strategies. ML and DL models have already demonstrated strong performance in cancer prediction, resistance profiling, and clinical decision support based on EV-derived signatures. However, clinical translation requires standardized EV isolation and characterization workflows, robust external validation, transparent and reproducible AI methodology, and prospective multicenter evaluation to ensure safety, reliability, and regulatory acceptance. The convergence of EV biology, multi-omics technologies, and AI-enhanced computational analysis has the potential to transform oncology by supporting minimally invasive diagnostics and truly personalized cancer therapy. Continued interdisciplinary collaboration and methodological rigor will be essential to realize the full clinical impact of EV–AI platforms in precision oncology.

## Figures and Tables

**Table 1:** EVs that are implicated in HCC pathogenesis [38,39,40,41,42,43,44,45,46,47,48,49,50,51,52,53,54,55,56,57,58,59,60,61,62,63,64,65,66,67,68,69,70,71,72,73,74,75,76,77,78,79,80,81,82,83,84,85,86,87,88,89].

EV Origin	Cargo	Clinical Significance	Reference
HCC cells	CD147	EMT via MMP-2 overproduction	[38]
	miR-92a-3p	EMT and metastasis via PI3K/AKT activation and PTEN suppression	[39]
	miR-21	HSC into CAF transformation promotes tumor progression, angiogenesis, EMT, drug resistance, and immune suppression	[40,41]
	miR-3129	EMT via targeting TXNIP; promotes metastasis	[42]
	lncRNAs MALAT1	Sponges miR-26a/b; promotes metastasis	[44]
	FAL1	Binds miR-1236; regulates ZEB1 gene expression and AFP	[45]
	TUC339	Uptake by CAFs promotes EMT, drug resistance, and metastatic dissemination Uptake by macrophages induces M2 phenotype, impaired phagocytosis, and tumor progression Most abundantly released EVs in HCC	[46]
	Vps4A	role as a tumor suppressor and regulator of exosome sorting, preventing the sorting of β-catenin and other tumor-promoting exosomal cargo. Loss of its function leads to HCC progression, and metastasis	[47]
	ATB	binds the miR-200 family and activates ZEB1/2 Promotes drug resistance, tumor progression, proliferation, migration, EMT, and metastasis	[48]
	ROR	Resistance to sorafenib via the activation of PI3K/AKT signaling pathway; it also activates the TGF-β pathway [65]	[49]
	miR-25	Resistance to sorafenib, promotes tumor progression, metastasis; it is highly expressed in metastatic HCC-derived EVs Activates the Wnt/β-catenin pathway by reducing SIK1 expression	[50]
	circ-PTGR1	Promotes tumor invasion and migration	[51]
	circFBLIM1	Tumor promotion, via its interaction with miR-338 and LRP6	[52]
	circ-0072088	Increases YAP1, leading to tumor cell proliferation and progression via sponging miR-375	[53]
	circ-0004001	Serves as a biomarker for disease progression and metastasis	[54]
	circ-0004003	Oncogenic effect via sponging tumor-suppressing miRNAs	[55]
	circ-0051443	Tumor suppressive effect via the sponging of miR-331-3p, leading to HCC apoptosis	[56]
	miR-429	Promotion of POU5F1 via targeting of RBBP4, leading to HCC progression	[57]
	circ-0051443	Increases the survival of HCC cells and suppresses their apoptosis	[58]
	miR-221	Downregulates p27/Kip1; promotes proliferation and NF-κB-mediated progression	[59,60]
	miR-23a	Suppresses NK cells and induces PD-L1 in macrophages, resulting in immune evasion	[61]
	let-7b	Uptake by TAMs induces cytokine release (e.g., IL-6)	[62]
	PD-L1	Suppresses T-cell response (suppression of the immune checkpoint); tumor escape phenomenon	[63]
	TGF-β	T-cell exhaustion, leading to suppressed cytotoxicity	[64]
	circ-UHRF1	Sponges miR-449c-5p, leading to immune evasion by suppressing NK function	[65]
	miR-103	Modifies endothelial cells; increases vascular permeability; promotes metastasis	[66]
	miR-210	Promotes neoangiogenesis under hypoxia	[67]
	H19	Promotes neoangiogenesis and fibrosis via VEGF/VEGFR	[68]
	VEGF	Received by ECs; promotes angiogenesis and metastasis	[69]
	CLEC3B	Tumor-suppressive cargo Downregulation or loss of its function leads to neoangiogenesis and metastasis	[70]
Adipocytes	miR-23a/b	Promotes HCC progression via miR-34a suppression	[71]
TAMs	miR-92a-2-5p	Activates AKT/β-catenin; suppresses androgen receptor → HCC progression	[72]
	miR-27a-3p	Promotes neoangiogenesis via HIF-1α/VEGF	[73]
	PD-L1	Suppresses CD8+ T-cells, leading to immune evasion	[74]
MSCs	lncRNA FENDRR	Tumor suppressive via β-catenin regulation	[75]
	miR-199a	Tumor suppressive; targets mTOR	[76]
	TRAIL	Induces HCC apoptosis	[77]
	miR-122	Increases chemotherapy sensitivity; tumor suppressive	[78]
CAFs	lncRNA-SNHG3	Activates TGF-β; promotes HCC progression	[80]
	miR-320a	Normally tumor suppressive; loss → HCC proliferation and metastasis	[81]
	miR-21-5p	Promotes migration and sorafenib resistance	[82]
	miR-20a-5p	Promotes EMT and tumor growth via PI3K/AKT activation	[83]
HSCs	TGF-β1	Activates SMAD signaling; fibrosis	[84]
	miR-335-5p	Tumor inhibitory via ROCK1 suppression	[85]
	miR-148-3p	Tumor inhibitory; downregulation → EMT, progression	[86]
Normal hepatocytes	SENP3-EIF4A1	Tumor suppressive via miR-9-5p targeting	[88]
Huh7 cell lines	miR-122	Tumor inhibitory via IGF-1 overexpression	[89]

AFP—alpha-fetoprotein; CAF—cancer-associated fibroblast; EC—endothelial cell; EMT—epithelial–mesenchymal transition; HIF-1α—hypoxia-inducible factor 1-alpha; HSC—hepatic stellate cell; IGF-1—insulin-like growth factor 1; LRP6—low-density lipoprotein receptor-related protein 6; MMP—matrix metalloproteinase; MSC—mesenchymal stem cell; NF-κB—nuclear factor kappa-light-chain-enhancer of activated B cells; NK—natural killer cell; PD-L1—programmed death-ligand 1; PI3K/AKT—phosphoinositide 3-kinase/protein kinase B pathway; PTEN—phosphatase and tensin homolog; ROCK1—Rho-associated protein kinase 1; TAM—tumor-associated macrophage; TGF-β—transforming growth factor beta; TXNIP—thioredoxin-interacting protein; VEGF—vascular endothelial growth factor; VEGFR—vascular endothelial growth factor receptor; YAP1—Yes-associated protein 1; ZEB1/2—zinc finger E-box-binding homeobox 1/2.

**Table 2:** EVs that are implicated in GBC pathogenesis [93,94,95,96,97].

EV Cargo	EV Source	Effect
miR-1246 [93]	Serum from GBC patients	Overexpressed in GBC; tumor-promoting cargo that enhances tumor progression and invasiveness; potential serum biomarker with CEA and CA19-9 (AUC = 0.816).
miR-451a [93]		Tumor-suppressive cargo; significantly decreased in GBC; inhibits proliferation by suppressing MIF, CDKN2D, and PSMB8, and promotes apoptosis.
THBS1 [94]	GBC cell line-	Glycoprotein in ECM; induces its modification and EMT, facilitating invasiveness via promoting TGF-β and integrin-triggered pathways (FAK, PI3K/AKT, MAPK/ERK, Src).
Haptoglobin [94]		Glycoprotein binding hemoglobin; elevated in GBC vs healthy/lithiasis (AUC = 0.826); associated with tumor growth, proliferation, and TME remodeling.
ANXA2 [94]		Calcium-dependent phospholipid-binding protein; implicated in invasiveness, migratory behavior, and metastatic dissemination via ECM modification.
pyruvate kinase M [94]		Involved in the Warburg effect; promotes proliferation and survival even under hypoxic or nutrient-deprived conditions.
ANPEP [94]		Metalloproteinase facilitates invasiveness, migration, and neoangiogenesis via activating MAPK and integrin-triggered pathways.
NT5E [94]		Alters anti-cancer immune response; suppresses NK and T-cell activity and promotes proliferation via adenosine overexpression in TME.
Neprilysin [94]		Implicated in invasion, proliferation, and neoangiogenesis via dysregulating EGFR and PI3K/AKT pathways.
Palmitic acid [97]	Bile	Tumor-promoting cargo; activates PI3K/AKT pathway.
Unsaturated phosphatidylethanolamines/phosphatidylcholines [97]		Reduced in GBC; associated with altered exosomal membrane integrity, aiding distinction from benign gallbladder pathologies.
miR-181c [97]		Overexpressed in GBC; tumor-promoting cargo.

ANPEP—aminopeptidase N; ANXA2—annexin A2; CA19-9—carbohydrate antigen 19-9; CDKN2D—cyclin-dependent kinase inhibitor 2D; CEA—carcinoembryonic antigen; ECM—extracellular matrix; EGFR—epidermal growth factor receptor; EMT—epithelial–mesenchymal transition; EV—extracellular vesicle; FAK—focal adhesion kinase; GBC—gallbladder cancer; MAPK/ERK—mitogen-activated protein kinase/extracellular signal-regulated kinase; miR—microRNA; MIF—macrophage migration inhibitory factor; NK—natural killer cell; NT5E—5′-nucleotidase ecto (CD73); PI3K/AKT—phosphoinositide 3-kinase/protein kinase B pathway; PSMB8—proteasome subunit beta type-8; Src—Src family kinases; TGF-β—transforming growth factor beta; TME—tumor microenvironment.

**Table 3:** EVs that are implicated in CCA pathogenesis [105,106,107,108,109,110,111,112,113,114,115,116,117,118,119,120,121,122,123,124,125,126,127,128,129,130,131,132,133,134,135,136,137,138,139,140].

EV Cargo	EV Source	Effect
miR-221	CCAs	Induces CCA proliferation, survival, aggressiveness; targets CDKN1B/p27 & PTEN; ↑ PI3K/AKT; biomarker [105,106].
miR-21		Activates IL-6/STAT3; suppresses PTEN & PDCD4; promotes invasiveness, EMT, drug-resistance; biomarker in plasma/bile [106,107,108].
miR-34c		Downregulated; targets WNT1; activates CAFs; linked to progression [109].
miR-30e		Downregulated; suppresses EMT; limits invasiveness & dissemination [110].
miR-26a		Targets KRT19; promotes growth; overexpression induces β-catenin via GSK3β [111].
miR-200 family		Increased; stage-related; induces EMT; modulates EC polarity; suppresses ZEB1/2; poor prognosis [112].
miR-183-5p		Overexpressed; induces neoangiogenesis (mast-cell VEGF); promotes progression & chemoresistance (PD-L1); ↑ PGE1/PGE2 [113].
circ-0000284		Sponges miR-637 → ↑ LY6E; malignant transformation; biomarker in plasma/bile [114].
circ-CCAC1		Cross-talk with ECs; neoangiogenesis; ↑ migration via miR-514a-5p → YY1; poor differentiation [115].
miR-192–5p		Overexpressed; regulates proliferation & apoptosis via MEK/ERK [116,117].
lncRNA-H19		Competes let-7 → ↑ HMGA2; malignant transformation; prognostic biomarker [118].
EpCAM		↑ Aggressiveness; promotes oncogenesis, migration, immune escape; predictive biomarker [119].
ctDNA		Gene mutations + methylated DNA; tool for minimal residual disease monitoring [120].
MUC1		Activates EGFR & β-catenin; ↑ invasiveness/migration; early biomarker (bile) [119].
Claudin-3 (CLDN3)		Loss of EC integrity; ↑ migration/metastasis; combined panel with EpCAM/MUC1 [121].
HER2		↑ Progression via MAPK/AKT; druggable (trastuzumab) [120,122].
Integrins, vitronectin, FZD10, lactadherin		Promote migration, metastasis, proliferation; ↑ β-catenin [123].
BMI1		Promotes growth, migration, metastasis; therapeutic target [124].
TNF-α, IL-6		Enhance inflammation & fibrosis via NF-κB/STAT3 [125].
Ceramide/dihydroceramide		Abundant; linked to metastasis & cytokine oversecretion [126].
LINC01812		Induces TAM M2-polarization; promotes perineural invasion [127].
miR-210		Overexpression ↓ RECK; ↑ growth, metastasis, chemoresistance [128].
EVs (M2 TAM)	TAMs	Suppress CD8+ T-cell cytotoxicity; immune escape [129].
VEGF		Promotes neoangiogenesis [129].
circ-0020256		Alters T-cell chemotaxis; ↑ proliferation, migration, metastasis [130].
miR-195	HSCs	Tumor-inhibiting; suppresses CCA cell lines [131].
miR-210	CAFs	Pro-oncogenic; enhances EMT & growth [132].
Chemokines (CCL2/5/7/8, CXCL12), PDGF, VEGF, M-CSF		Recruit TAMs; promote CCA progression [129,133].
HCV proteins/RNA	Infected host-cells	Chronic inflammation, oxidative stress; activates IL-6/STAT3, NF-κB, JAK/STAT3, MAPK; fibrosis; EMT; biomarker for iCCA [134].
HBV proteins/DNA		Immune evasion; EMT, oxidative stress; EV-HBx causes DNA methylation; early biomarker for CCA/HCC [4,135,136].
Endotoxin EVs	Dysbiotic microbiome	Activate TLR4/NF-κB; inflammation/oncogenesis [137].
Vimentin, CCL2, CXCL1, α-SMA, FAP	HuCCT1cell lines	Promote progression [138].
Csi-let-7a-5p	*C. sinensis*	Downregulates PTEN, SOCS1, PRDM1; activates AKT/mTOR & JAK2/STAT3; modulates M1 macrophages [139].
EVs (multiple cargos)	*O. viverrini*	Alter MAPK, ↑ IL-6, impair wound repair; promote cholangiocarcinogenesis [140].

α-SMA—Alpha-Smooth Muscle Actin; AKT—Protein Kinase B; BMI1—B-cell-specific Moloney murine leukemia virus Integration site 1; CAF—Cancer-Associated Fibroblast; CCA—Cholangiocarcinoma; CLDN3—Claudin-3; ctDNA—Circulating Tumor DNA; EC—Endothelial Cell; EpCAM—Epithelial Cell Adhesion Molecule; FAP—Fibroblast Activation Protein; FZD10—Frizzled Class Receptor 10; HBx—HBV X Protein; HCV—Hepatitis C Virus; HER2—Human Epidermal Growth Factor Receptor 2; HSC—Hepatic Stellate Cell; iCCA—Intrahepatic Cholangiocarcinoma; IL—Interleukin; JAK—Janus Kinase; MAPK—Mitogen-Activated Protein Kinase; MUC1—Mucin 1; NF-κB—Nuclear Factor kappa-light-chain-enhancer of activated B cells; PDCD4—Programmed Cell Death Protein 4; PGE2—Prostaglandin E2; PTEN—Phosphatase and Tensin Homolog; SOCS1—Suppressor of Cytokine Signaling 1; STAT—Signal Transducer and Activator of Transcription; TLR4—Toll-Like Receptor 4; TNF—Tumor Necrosis Factor; TAM—Tumor-Associated Macrophage; VEGF—Vascular Endothelial Growth Factor; YY1—Yin Yang 1; ZEB—Zinc Finger E-box-binding Homeobox; mTOR—Mechanistic Target of Rapamycin; ↓ decrease; ↑ increase.

**Table 4:** EVs implicated in PDAC progression [143,144,145,146,147,148,149,150,151,152,153,154,155,156,157,158,159,160,161,162,163,164,165,166,167,168,169,170,171,172,173,174,175,176,177,178,179,180,181,182].

EV Source	Cargo	Effect
PDAC	KRAS [143]	Increased in PDAC; prognostic biomarkers correlated with PDAC progression and poor survival
	EGFR [144]	Increased in PDAC development and progression
	CD44 [145]	Increased in PDAC; correlated with disease progression
	miR-222 [146]	Increased in PDAC; correlated with stage and tumor dimension
	miR-27a [147]	Increased, correlated with invasion, metastasis, and neoangiogenesis via BTG2 suppression; potential therapeutic target
	CKAP4 [148]	Increased, especially preoperatively; correlated with progression via Wnt pathway
	miR-125b-5p [149]	Implicated in PI3K/Akt/FoxO1 and glucose regulation; promotes insulin resistance, invasiveness, EMT via MEK/ERK
	miR-197-3p [150]	Implicated in GIP and GLP-1 expression
	miR-19a [151]	Implicated in Neurod1 expression; affects β-cell regulation and PDAC-related DM
	miR-3148/miR-3133/miR-144-5p [152]	Implicated in DM or insulin intolerance via altering islet cells
	miR-125b-5p [153]	Promotes PDAC invasiveness and metastatic dissemination, as well as EMT, via overregulation of the MEK/ERK pathway
	miR-6796-3p [150]	Implicated in glucose metabolism via modifying GIP and GLP-1
	miR-155 [154]	Highly found; suppresses apoptosis; promotes survival and chemoresistance
	CAV1 [155,156]	Promotes proliferation; suppresses apoptosis; prognostic variability
	O-glycan-binding lectin [157]	Increased preoperatively; decreased postoperatively
	miR-4750-3p [150]	Implicated in glucose dysregulation
	ITGA3 [150]	Implicated in ECM modification
	miR-6763-5p [150]	Implicated in glucose dysregulation
	ITGΒ5 [145]	Implicated in PDAC cell adhesion
	miR-450b-3p [158]	Implicated in glucose dysregulation and insulin intolerance via PI3K/Akt/FoxO1
	B2M [159]	Increased; promotes escape from tumor surveillance
	PODX [145]	Implicated in invasiveness and migration
	miR-666-3p [158]	Implicated in insulin resistance via PI3K/Akt/FoxO1
	S100A4 [145]	Promotes motility and migration
	STAT14 [145]	Promotes metastatic dissemination
	miR-883b-5p [158]	Promotes insulin resistance
	F3 [145]	Takes part in immune cell recruitment
	LAMP1 [145]	Promotes metastatic dissemination
	miR-540-3p [158]	Induces insulin intolerance via PI3K/Akt/FoxO1
	ANXA1 [145]	Enhances inflammation; suppresses apoptosis
	Lin28B [160]	Promotes metastatic dissemination
	Adrenomedullin [161]	Potential marker for PDAC-related DM; correlated with β-cell destruction
	Integrins [159]	Induce stromal modification promoting invasion, migration, dissemination
	GPC1 [162]	Highly found; correlated with decreased survival; debated diagnostic value
	miR-21 [163,164]	Highly upregulated; related to chemoresistance; promotes invasion, dissemination, apoptosis inhibition
	ZIP4 [165]	High levels correlate with highly aggressive PDAC; therapeutic target
	MIF [166]	Promotes pre-metastatic niche; associated with PDAC stage; induces TGFβ and fibronectin
	Tspan8 [167]	Promotes motility, migration, metastatic dissemination
	EphA2 [168]	Increased, especially in advanced tumors; associated with favorable neoadjuvant response
	CD151 [169]	Implicated in stromal modification; associated with KRAS mutations
	Pre-metastatic niche proteins [170]	Implicated in pre-metastatic niche and distant hyperpermeability
	CLDN1 [171]	Related to poor prognosis
	CLDN4 [172]	Related to KRAS mutations
	MUC1 [144,173]	Related to unfavorable prognosis
	HIST2H2BE [172]	Related to the KRAS mutation
	EpCAM/LGALS3BP [169]	Related to KRAS mutation
Adipocytes	Lipids, FA oxidation enzymes [174]	Provide metabolic support; promote PDAC progression (especially in obesity)
PSC	TGF-β proteins [175]	Implicated in ECM modification and EMT
	miR-21-5p/miR-451a [176]	Promote ECM modification, progression, invasion
	miR-5703 [176]	Promotes proliferation via PI3K/Akt and CMTM4 suppression
CAFs	Metabolites [177]	Promote tumor progression under nutrient lack
	miR-21 [178]	Upregulated; related to chemoresistance and unfavorable prognosis
	miR-146 [179]	Promotes immune suppression, proliferation, chemoresistance
	ANXA6 [180]	Implicated in pre-metastatic niche formation
	miR-155 [181]	Promotes survival and chemoresistance
M2 macrophages	miR-155-5p/miR-221-5p [182]	Promote neoangiogenesis, growth, proliferation via E2F2

PDAC (Pancreatic Ductal Adenocarcinoma), EV (Extracellular Vesicle), KRAS (Kirsten Rat Sarcoma Viral Oncogene Homolog), EGFR (Epidermal Growth Factor Receptor), CD44 (Cluster of Differentiation 44), miR (MicroRNA), BTG2 (B-cell Translocation Gene 2), CKAP4 (Cytoskeleton-Associated Protein 4), PI3K (Phosphatidylinositol 3-Kinase), Akt (Protein Kinase B), FoxO1 (Forkhead Box O1), GIP (Glucose-Dependent Insulinotropic Peptide), GLP-1 (Glucagon-Like Peptide-1), DM (Diabetes Mellitus), EMT (Epithelial–Mesenchymal Transition), MEK (Mitogen-Activated Protein Kinase Kinase), ERK (Extracellular Signal-Regulated Kinase), CAV1 (Caveolin-1), ITGA3 (Integrin Alpha 3 Subunit), B2M (Beta-2 Microglobulin), PODX (Podocalyxin-Like Protein), TGFβ (Transforming Growth Factor Beta), ANXA1 (Annexin A1), ZIP4 (Zinc Transporter 4), MIF (Macrophage Migration Inhibitory Factor), Tspan8 (Tetraspanin 8), EphA2 (Ephrin Type-A Receptor 2), CD151 (Cluster of Differentiation 151), CLDN1 (Claudin-1), CLDN4 (Claudin-4), MUC1 (Mucin 1), HIST2H2BE (Histone Cluster 2 H2B Family Member E), EpCAM (Epithelial Cell Adhesion Molecule), LGALS3BP (Galectin-3-Binding Protein), FA (Fatty Acid), ECM (Extracellular Matrix), CAF (Cancer-Associated Fibroblast).

**Table 5:** EV isolation and detection methods—pros and cons of EV utilization [119,235,236,237,238,239,240,241,242].

Isolation/Detection Methods	Advantages	Limitation
Isolation Ultracentrifugation (UC)	Ideal for exosome isolation (~100,000× *g*)	Time-consuming; may compromise EV integrity possible contamination (e.g., lipoproteins and protein aggregates)
Size Exclusion Chromatography (SEC)	Gentle; preserves EV integrity and functionality; highly purified EVs; useful for large oncosomes in cancer studies	Not suitable for large-volume samples compared to UC
Density Gradient Ultracentrifugation (DGUC)	Provides more purified EVs than UC	Complex; time-consuming
Polymer-Based Precipitation (PBP)	Simple, fast, and does not require special equipment	Low purity due to contamination
Immunoaffinity Capture (IAC)	Highly selective for EVs; isolates subpopulations based on surface markers	Low yield; limited by cost; may miss EVs lacking target markers
Microfluidics-Based Isolation (MBI)	Separate EVs based on size, surface markers, or charge; potential for clinical applicability	Requires specialized lab-on-a-chip devices; not widely available
Ultrafiltration (UF)	Fast and easy; can be combined with other methods	Shear forces may alter EV integrity
Acoustic Fractionation/Field-Flow Separation (FFS)	Can isolate EVs based on acoustic or electromagnetic properties	Complex; not widely studied or used
Detection/Characterization		
Flow Cytometry (FC)	Can detect large oncosomes; allows use of antibody-coated beads	Not suitable for particles < 500 nm
Nanoparticle Tracking Analysis (NTA)	Provides size, concentration, and distribution information	Not widely available in clinical labs; cannot analyze particles > 400 nm quantitatively
EV Imaging (EM, Optical/Confocal, IF, IHC)	Visualizes exosomes, microvesicles, and large oncosomes; allows structural and localization studies	Requires specialized equipment; labor-intensive
Nanoplasmonic Fluorescence-Amplified EV Sensing Technology (FLEX assay).	A plasmonic gold-nanowell chip that captures tumor-derived EVs	FLEX detects small tEVs, which are impossible to detect by conventional EV fluorescence imaging

UC: Ultracentrifugation; SEC: Size Exclusion Chromatography; DGUC: Density Gradient Ultracentrifugation; PBP: Polymer-Based Precipitation; IAC: Immunoaffinity Capture; MBI: Microfluidics-Based Isolation; UF: Ultrafiltration; AF/FFS: Acoustic Fractionation/Field-Flow Separation; FC: Flow Cytometry; NTA: Nanoparticle Tracking Analysis; EM: Electron Microscopy; IF: Immunofluorescence; IHC: Immunohistochemistry; FLEX: Nanoplasmonic fluorescence-amplified EV sensing.

**Table 6:** Biological, technical, and translational challenges in AI-driven EV-based oncology research. These challenges reflect intrinsic properties of EV biology and clinical translation rather than inherent limitations of artificial intelligence. Their correct identification and mitigation require expert-level methodological design, appropriate validation strategies, and interdisciplinary integration of EV biology, clinical oncology, and advanced AI modeling.

Challenge Domain	Limitations
Methodological heterogeneity	EV studies differ in experimental design and analytical workflows due to biological complexity and evolving technologies; this requires expert computational modeling and rigorous validation rather than indicating unsuitability of AI.
Technical variability	Variability in EV isolation, molecular profiling, and measurement platforms introduces technical signal that can confound biological interpretation if not handled with domain-aware modeling strategies.
EV standardization and reproducibility	Differences in isolation protocols, quantification methods, and annotation standards generate batch effects that must be addressed through careful experimental control and expert model design rather than post hoc statistical harmonization alone.
EV isolation and contamination	Common isolation methods co-isolate lipoproteins and protein aggregates, and no current approach removes 100% of non-vesicular particles (MISEV2023), limiting biological specificity of EV measurements.
EV heterogeneity	EV subpopulations vary by cellular origin, disease state, and microenvironment, complicating interpretation of bulk EV signatures, especially in patients with multimorbidity or overlapping disease processes.
Detection and characterization variability	Heterogeneous detection and imaging platforms (e.g., NTA, flow cytometry, TEM) yield partially non-overlapping EV measurements, reducing cross-study comparability.
Storage and stability	Freeze–thaw cycles and storage conditions can alter EV integrity and cargo composition, particularly for RNA species, introducing additional biological noise.
Manufacturing constraints	High production costs, low EV yields, and scalability limitations restrict clinical translation of EV-based diagnostics and therapeutics.
Incomplete biological understanding	EV uptake mechanisms, biodistribution, and functional cargo selection remain only partially understood, limiting mechanistic interpretability of EV biomarkers.
Risk of learning technical artifacts	In the absence of appropriate experimental control and expert model design, AI systems may learn protocol-dependent features rather than disease biology, leading to spurious biomarker discovery.
Limited datasets and cohort diversity	Many EV datasets remain small and geographically or etiologically restricted, increasing the importance of expert validation strategies (e.g., nested cross-validation, external testing) to avoid overfitting and population bias.
Model interpretability	Black-box models hinder mechanistic insight and regulatory acceptance unless paired with explainable AI approaches that identify biologically meaningful EV features.
Multimodal data integration	EV-omics, imaging, and clinical variables are often analyzed separately, limiting biological insight into tumor evolution and treatment response unless advanced fusion architectures are employed.
Clinical validation	Most EV–AI studies remain retrospective; prospective evaluation is required to establish clinical utility rather than only statistical performance.
Regulatory and safety considerations	EV–AI systems must satisfy evolving digital health and biomanufacturing standards, including auditability, robustness testing, and post-deployment monitoring.
Equity and generalizability	Models trained on restricted populations may not generalize across diverse healthcare settings without fairness-aware learning and international benchmarking.

AI—artificial intelligence; EV—extracellular vesicle.

## Data Availability

No new data were created or analyzed in this study. Data sharing is not applicable to this article.

## Abbreviations

AI	Artificial intelligence
AFP	Alpha-fetoprotein
ARRDC1	Arrestin domain-containing protein 1
ARF6	ADP-ribosylation factor 6
BDNF	Brain-derived neurotrophic factor
CAF	Cancer-associated fibroblast
CCA	Cholangiocarcinoma
circRNA	Circular RNA
CRC	Colorectal cancer
CXCL12	C-X-C motif chemokine ligand 12
DNA	Deoxyribonucleic acid
EC	Endothelial cell
ECM	Extracellular matrix
EM	Electron microscopy
EMT	Epithelial–mesenchymal transition
ERK	Extracellular signal-regulated kinase
ESCRT	Endosomal sorting complex required for transport
EV	Extracellular vesicle
GC	Gallbladder cancer
GI	Gastrointestinal
HCC	Hepatocellular carcinoma
HDI	Human development index
HIF-1α	Hypoxia-inducible factor 1 alpha
HSC	Hepatic stellate cell
HUVEC	Human umbilical vein endothelial cell
IFN-γ	Interferon gamma
IL	Interleukin
ILV	Intraluminal vesicle
lncRNA	Long non-coding RNA
LO	Large oncosome
LRP6	Low-density lipoprotein receptor-related protein 6
MAPK	Mitogen-activated protein kinase
MASLD	Metabolic dysfunction-associated steatotic liver disease
miRNA (miR)	MicroRNA
MMP	Matrix metalloproteinase
MSC	Mesenchymal stem cell
MV	Microvesicle
MVB	Multivesicular body
mTOR	Mechanistic target of rapamycin
NF-κB	Nuclear factor kappa B
NK cell	Natural killer cell
NTA	Nanoparticle tracking analysis
PC	Pancreatic cancer
PD-1	Programmed cell death protein 1
PD-L1	Programmed death-ligand 1
PI3K	Phosphoinositide 3-kinase
PLS-DA	Partial least squares discriminant analysis
PTEN	Phosphatase and tensin homolog
Rab	Ras-related small GTPase
RF	Random Forest
RNA	Ribonucleic acid
ROCK1	Rho-associated coiled-coil-containing protein kinase 1
SERS	Surface-enhanced Raman spectroscopy
SEC	Size exclusion chromatography
sEV	Small extracellular vesicle
SMAD	SMAD family protein
SNARE	Soluble NSF-attachment protein receptor
STAT	Signal transducer and activator of transcription
TAM	Tumor-associated macrophage
tEV/tEVs	Tumor-derived extracellular vesicle(s)
TGF-β	Transforming growth factor beta
TRAIL	TNF-related apoptosis-inducing ligand
TEM	Transmission electron microscopy
TSG101	Tumor susceptibility gene 101
TME	Tumor microenvironment
UC	Ultracentrifugation
VAMP	Vesicle-associated membrane protein
VEGF	Vascular endothelial growth factor
VEGFR	Vascular endothelial growth factor receptor
Vesiclepedia	EV-focused database
XAI	Explainable artificial intelligence
